# Early-Life Red Light Exposure Extends Lifespan in *C. elegans* through ROS-AMPK-Driven Mitochondrial Reprogramming

**DOI:** 10.14336/AD.2025.0791

**Published:** 2026-09-09

**Authors:** Yao Zhu, Xin Lin, Huiting Wang, Mingming Song, Tao Yang, Yi Jiao, Chang Liu

**Affiliations:** ^1^Department of Endocrinology, Nanjing Drum Tower Hospital, China Pharmaceutical University, Nanjing 211198, China.; ^2^Department of Biochemistry and Molecular Biology, School of Basic Medical Sciences, Xinjiang Medical University, Urumqi 830011, China.; ^3^State Key Laboratory of Natural Medicines and School of Life Science and Technology, China Pharmaceutical University, Nanjing 211198, China.; ^4^Jiangsu Provincial University Key Laboratory of Drug Discovery for Metabolic Inflammatory Diseases, China Pharmaceutical University, Nanjing 211198, China.; ^5^School of Pharmaceutical Sciences, Anhui Medical University, Hefei 230032, China.; ^6^The Fourth Affiliated Hospital of Nanjing Medical University, Nanjing 211198, China.

**Keywords:** *C. elegans*, Light photobiomodulation, Mitochondrial quality control, ROS-AMPK axis, Lifespan

## Abstract

Specific wavelengths and intensities of light offer potential for modulating mitochondrial function to influence aging which is important for the development of non-invasive and modifiable exogenous anti-aging tools. However, the effects of exposure to different wavelengths of light during the early stages of life on health in adulthood, and the underlying mechanisms, remain poorly understood. In this study, we utilized *Caenorhabditis elegans* to investigate the effects of early-life (L1 to young adult stage) exposure to different light wavelengths (white, red, blue, green) on healthspan. We found that red light exposure (630 nm, 200 lx) during early life extended lifespan by 10.34% without affecting growth, movement, or reproduction, and improved late-life health indicators. Importantly, it significantly enhanced healthspan in later life, suggesting its potential as a non-invasive anti-aging strategy. Mechanistically, red light transiently suppressed mitochondrial bioenergetic activity, biogenesis, and membrane potential, and altered mitochondrial dynamics. Upon light withdrawal, mitochondrial function progressively recovered, demonstrating enhanced structure and function by day 6. Furthermore, red light stimulated ROS production, activated AMPKα phosphorylation, and triggered downstream mitochondrial quality control (MQC) programs, including the unfolded protein response (UPR^MT^) and mitophagy. Notably, these protective effects were conserved in human dermal fibroblasts, suggesting the translational applicability of this pathway. Collectively, our study identifies a specific “photophysiological window” during early life through which red light promotes longevity via ROS-mediated activation of AMPK and enhancement of MQC, proposing a non-invasive, safe, evolutionarily conserved, and drug-free strategy to mitigate age-related mitochondrial decline. Therefore, red light can serve as a non-invasive anti-aging strategy to enhance healthspan and potentially alleviate age-related mitochondrial dysfunction.

## INTRODUCTION

With the rapid advancement of public health and medicine, global population aging is accelerating, with projections suggesting that individuals aged 65 and above will comprise between 16 and 22% of the global population by 2050 [1]. This demographic shift is expected to increase the burden of age-related diseases, including cardiovascular diseases, metabolic syndrome, and neurodegenerative disorders [2]. In response, proactive health management and early intervention strategies are increasingly prioritized to promote healthy aging, underscoring the need for lifespan-wide anti-aging approaches in public health initiatives [3].

The aging process is influenced by multiple internal and external factors, and its underlying mechanisms involve oxidative stress, metabolic imbalance, genomic instability and mitochondrial dysfunction [4]. Although several anti-aging intervention strategies have been extensively explored and validated in a variety of model organisms, including lifestyle interventions such as dietary restriction, exercise, and temperature stimulation, as well as small-molecule drugs targeting mTOR, sirtuins, and other pathways, their practical application remains limited due to off-target effects, safety concerns, and issues with compliance [5-8]. As a non-drug, non-invasive, temporally as well as spatially precise controllable intervention, light emerges as a crucial regulator in modulating aging and longevity [9]. Notably, the application of a twice-weekly LED mask has demonstrated significant potential in reversing skin aging [10]. Furthermore, Short-term laser stimulation has been shown to enhance protein synthesis and optimizes energy metabolism [11, 12]; near-infrared light (670 nm) has been reported to promote retinal energy metabolism and alleviates inflammation in aged mice [13], whereas green light irradiation exhibited potential to delay aging in Drosophila [14]. These findings suggest that specific wavelengths of light act as critical modulators that help ameliorat tissue function and decelerating aging processes.

In contrast to visually dependent organisms, the model organism *Caenorhabditis elegans*, representing a typical non-visual model organism, offers several unique advantages, including a short life cycle, well-defined developmental synchronization, a clear genetic background, and high sensitivity to environmental stimuli. These characteristics have established it as an indispensable tool in aging and photobiology research [15]. Previous studies have shown that ultraviolet light triggers nematode escape responses, specific light wavelengths modulate nematode foraging behaviors, and prolonged exposure to visible light reduces lifespan. However, the majority of current research on light exposure in nematodes has focused on full-life-cycle continuous exposure or short-term laser stimulation, primarily evaluating acute photo-oxidative stress responses and their short-term impacts on lifespan or behavior [16, 17]. Despite these findings, a significant gap remains in understanding the impact of targeted light interventions during critical windows of early life, as well as their potential implications for long-term health outcomes.

Although photobiomodulation (PBM) has been widely reported to optimize energy metabolism and reduce oxidative stress through the modulation of mitochondrial function[18, 19], the optimal timing of such interventions across the lifespan and the underlying mechanisms remain unclear. Developmental studies suggest that brief stimuli during early life can elicit long-term functional remodeling, indicating a high degree of physiological plasticity during this period [20]. Based on these insights, we introduce the concept of a "photophysiological window", a defined early-life period during which light exposure can induce durable effects on aging trajectories and homeostatic regulation through physiological reprogramming. This concept is inspired by the principle of mitohormesis, wherein transient, moderate mitochondrial stress triggers adaptive responses that enhance cellular resilience and extend the organismal lifespan [21].

Extensive evidence indicates that near-infrared (NIR) light can target the mitochondrial respiratory chain, enhancing ATP production while moderately elevating reactive oxygen species (ROS) levels [22]. Elevated ROS, in turn, can activate downstream AMPK signaling, a key regulator of cellular stress responses and energy homeostasis [23]. Concurrently, mitochondrial quality control (MQC) processes—including mitochondrial dynamics, autophagy (particularly mitophagy), and the mitochondrial unfolded protein response—play central roles in the regulation of aging [24]. Although these pathways have been associated with lifespan extension across various models, whether they act as downstream effectors of the ROS-AMPK axis in mediating responses to light exposure remains unclear.

Based on these insights, we propose the concept of “photophysiological window,” utilizing *C. elegans* as a model system to comprehensively assess the impact of specific light wavelengths during early life on lifespan regulation and long-term health trajectories. In this study, we demonstrate that early-life red light exposure significantly prolongs lifespan and enhances late-life health indicators in nematodes. Furthermore, it strengthens the mitochondrial quality control network and promotes mitochondrial homeostasis remodeling activation of the ROS-AMPK axis. This phenomenon was also observed in human fibroblasts, indicating strong evolutionary conservation and substantial translational potential. Overall, this study provides a theoretical basis and practical foundation for developing non-pharmacological anti-aging strategies centered on early life-cycle interventions.

### MATERIALS AND METHODS

*C. elegans* strains and Maintenance

The *C. elegans* strains were obtained from the Caenorhabditis Genetics Center (CGC), University of Minnesota, USA. All experiments were performed using hermaphroditic worms, with the N2 strain serving as the wild-type control. Strains were maintained at 20 °C on nematode growth medium (NGM) plates seeded with *E. coli* strain OP50. Detailed strain information is provided in Supplementary Table 1. Synchronized populations were prepared by bleaching gravid adults, followed by overnight incubation in M9 buffer to allow hatching. Synchronized L1 larvae were cultured on 60 mm NGM agar plates (containing 3 g/L NaCl, 17 g/L agar, 2.5 g/L peptone, 1 mM CaCl_2_, 5 mg/L cholesterol, 1 mM MgSO_4_, and 25 mM KH_2_PO_4_/K_2_HPO_4_), seeded with OP50 *E. coli*.

### Lifespan Analysis

Synchronized L1 larvae were seeded on NGM plates seeded with OP50 *E. coli.* At the L4 stage, 30-60 worms were transferred to 35 mm NGM plates containing 10 μg/mL 5-fluoro-2′-deoxyuridine (FUDR). Starting from adulthood, worms were transferred to fresh plates every two days, and survival was recorded daily until death. Death was confirmed by the absence of a response to gentle stimulation with a sterilized platinum wire. Worms that burrowed into the agar, adhered to plate walls, or died from non-aging causes (e.g., internal hatching or vulval rupture) were censored. All experiments were conducted at 20 °C unless otherwise specified. Survival curves were generated using GraphPad Prism, with details provided in Supplementary Table 2 for lifespan. All lifespan experiments included three biological replicates.

### Brood Size Measurement

Total brood size was measured by individually plating late L4 worms exposed to different light wavelengths. Worms were transferred to fresh plates every 12 h, and the previous plates were kept in the dark at 20 °C for 48 h to allow progeny development. The number of live progeny (L3-L4 larvae) was then counted. This process was repeated until no live progeny was observed for four consecutive 48 h intervals. Brood size was assessed in nine individual worms per light condition.

### Measurement of Age Pigment Level

Worms were mounted on slides and anesthetized with 5 mg/mL levamisole. Intestinal autofluorescence was visualized using a fluorescence microscope (Nikon, Tokyo, Japan). Fluorescence intensity was quantified using ImageJ software and normalized to the body area.

### Measurement of Body Length and Surface Area

Body length and surface area of young adult worms were measured under different light conditions starting from hatching. After light exposure, worms were anesthetized with levamisole (5 mg/mL), aligned on unseeded NGM plates, and imaged under a microscope. Measurements were quantified using ImageJ software. A total of 20 individual worms were analyzed per condition.

### Locomotion Assay

Randomly selected nematodes were transferred to 2 mL of M9 buffer in a 35 mm culture dish. Body bends and thrashes were recorded over 30-second intervals using a dissecting microscope equipped with a camera attachment. A minimum of 30 individual nematodes was analyzed per treatment.

### Pumping Rate Assay

Pumping rates were measured by counting the number of contractions of the pharyngeal terminal bulb over 30-second intervals during light exposure on an OP50 E. coli bacterial lawn, as previously described. A single contraction of the posterior bulb or grinder was defined as one pump. Approximately 20 individual worms were analyzed per group.

### Detection of Nematode Pharyngeal Degeneration

Synchronized L1 larvae were exposed to light conditions until reaching the young adult stage. After light exposure, adult worms were washed three times with M9 buffer, anesthetized with levamisole (5 mg/mL), and imaged for pharyngeal degeneration under a microscope. Degeneration was scored on a five-point scale, where score 1 represented a young pharynx and scores 2-5 indicated progressively worsening degeneration [25].

Class 1 (young/intact): no or minimal vacuolation; patent lumen along >90% of tract; continuous pharyngeal muscle and basement membrane.

Class 2 (mild): few small vacuoles and mild isthmus narrowing; lumen largely patent; muscle continuity preserved.

Class 3 (moderate): increased vacuolation and focal muscle discontinuities; lumen constriction affecting a substantial segment; partial architectural distortion.

Class 4 (marked): extensive vacuolation with diffuse muscle discontinuity; segmental lumen collapse; multiple focal disruptions of the basement membrane.

Class 5 (severe): loss of pharyngeal structural integrity with widespread fragmentation; lumen collapse over large portions of the tract.

### Measurement of Body-Cavity Leakage

Body-cavity leakage was assessed using erioglaucine disodium salt (Sigma, Cat# 3844-45-9). A 10 % (w/v) solution of erioglaucine disodium salt was prepared in M9 buffer and mixed at a 1:1 ratio with OP50 *E. coli* bacterial culture. Worms were incubated in the mixture for 6 h, then washed three times with M9 buffer. Fluorescence images were acquired using a fluorescence microscope (Nikon, Tokyo, Japan). Leakage severity was categorized into three classes:

Class 1 (low): minimal or no leakage signal.

Class 2 (moderate): detectable localized fluorescence leakage.

Class 3 (high): widespread or intense fluorescence leakage throughout the body cavity.

### Real-time quantitative PCR (RT-qPCR)

Total RNA was extracted from nematodes using TRIzol™ reagent (*AG*, Cat# AG21101, China), and RNA concentration was measured with a NanoDrop spectrophotometer. One microgram (1 μg) of RNA was reverse-transcribed into cDNA. Quantitative PCR (qPCR) was performed using SYBR Green Mix (Accurate Biology, Cat# AG11701) on a LightCycler® 480 II (Roche). Relative gene expression was calculated using the 2^^-ΔΔCt^ method.

For *C. elegans*, mitochondrial copy number was estimated by the ratio of *nd-1* to *act-3*. For HFF-1 cells, *SLCO2B1*served as the nuclear DNA reference, and *ND1* as the mitochondrial marker. Detailed Primer sequence information is provided in Supplementary Table S3.

### Mitochondrial Stress Assay

Basal and maximal oxygen consumption rates (OCR) were measured using the Seahorse XF96/XF94 Analyzer (*Agilent Technologies*, USA). Nematodes were washed three to five times with M9 buffer to remove surface bacteria. Approximately 20 nematodes in 20 μL of S-basal solution were transferred to each well of a Seahorse XF96 Cell Culture Microplate containing 80 μL of 1× M9 buffer.

Basal OCR was recorded, followed by sequential injections through the reagent ports of dicyclohexylcarbodiimide (DCCD; ATP synthase inhibitor, 2 mM) and carbonyl cyanide-p-trifluoro- methoxyphenylhydrazone (FCCP; mitochondrial uncoupler, 1.25 mM) [26]. Mitochondrial function parameters were assessed, including basal OCR; ATP-linked respiration (basal OCR minus DCCD-inhibited OCR); maximal OCR (FCCP-induced OCR) and spare respiratory capacity (FCCP-induced OCR minus basal OCR). All measurements were conducted at 20-22 °C in three independent chambers for each condition and normalized to the number of nematodes per well.

### RNA interference experiments (RNAi)

RNAi experiments were conducted as previously describe [27]. Plasmids targeting *atfs-1* and *ubl-5* were provided by Dr. Dayong Wang’s lab (Southeast University, China). The RNAi clone for *lin-65* was constructed in-house using the following primers: forward 5'-caggaattcgatatcaagctt CAATGGTCACTCAGCCTACGACT-3' and reverse 5'-ctatagggcgaattgggtaccATCCTGTGCACTCGAATGAATCG-3'. Bacterial cultures were grown overnight in LB medium supplemented with 100 μg/mL ampicillin and 12.5 μg/mL tetracycline. OP50 cultures were then plated onto NGM plates containing 25 μg/mL carbenicillin and 1 mM isopropyl-β-D-1-thiogalactopyranoside (IPTG). Bacteria carrying the empty L4440 vector served as the control. Synchronized L1-stage worms (approximately 100 per plate) were transferred to RNAi plates and cultured to adulthood. To enhance knockdown efficiency, worms were passaged once, and the F1 progeny was used for subsequent experiments.

### Measurement of ATP content

ATP content was measured using the Enhanced ATP Assay Kit (*Beyotime*, Cat# S0026, China). Nematodes were washed three times with M9 buffer, resuspended in ATP lysis buffer (100-200 μL per 20 mg tissue), and lysed via ice-cold ultrasonication. Samples were centrifuged at 12,000 × g for 5 min at 4 °C, and the supernatant was collected. Luminescence was measured with a microplate reader (SpectraMax® iD3, Molecular Device, USA). Protein concentration was determined using the bicinchoninic acid (BCA) assay, and ATP content was normalized to protein concentration.

### Transmission Electron Microscopy (TEM) Analysis

After 60 h of light exposure, nematodes were washed three to five times with M9 buffer until the supernatant was clear. Samples were initially fixed in 2.5 % glutaraldehyde for 2-3 min, followed by removal of heads and tails using a microsurgical blade. Specimens were then fixed in fresh glutaraldehyde for 24 h at 4 °C. Ultrastructural analysis of mitochondria was performed using a transmission electron microscope.

### Measurement of Mitochondrial Membrane Potential

Mitochondrial membrane potential in C. elegans was assessed using the TMRE Mitochondrial Membrane Potential Assay Kit (*Beyotime*, Cat# C2001S, China). TMRE was dissolved in OP50 and spread onto NGM agar plates. Worms were incubated on TMRE-containing plates for 12 h. Fluorescence images were captured using a fluorescence microscope (Olympus, Japan), and fluorescence intensity was quantified using ImageJ software, normalized to body area.

For mammalian cells, after light exposure, cells were washed with PBS, and 1 mL of fresh medium supplemented with 1 mL of JC-10 working solution (Solarbio, Cat# CA1310-100, China) was added per well. Cells were incubated for 30 min at 37 °C prior to fluorescence detection.

### Western Blot Analysis

Western blot analysis was performed on lysates from ≥ 500 nematodes per group. Worms were washed with M9 buffer to remove surface bacteria, lysed in RIPA buffer with PMSF and phosphatase inhibitors by sonication, and centrifuged at 13,000 rpm for 15 min at 4 °C. Protein concentrations were determined using the bicinchoninic acid (BCA) assay (*Vazym*e, E112-01/02). Samples were boiled, separated by SDS-PAGE, and transferred to PVDF membranes. After blocking with 5 % non-fat milk, membranes were incubated with primary antibodies at 4 °C overnight, followed by HRP-conjugated secondary antibodies. Signals were detected via chemiluminescence (Tanon, China), and band intensities were quantified using ImageJ and normalized to GAPDH. The following antibodies were used: GAPDH (Cat# T0004, RRID: AB_2833041, Affinity Biosciences, USA); β-actin (Cat# AC038, RRID: AB_2863784, ABclonal, China); β-tubulin (Cat# A12289, RRID: AB_2861647, ABclonal, China); MTCO1 (Cat# A24805, RRID: AB_2861744, ABclonal, China); ATP5A1 (Cat# A11217, RRID: AB_2861524, ABclonal, China); CLPP (Cat# A9127, RRID: AB_2768958, ABclonal, China); PGC1α (Cat# A19674, RRID: AB_2862726, ABclonal, China); AMPKα (Cat# 2532S, RRID: AB_330331, Cell Signaling Technology, USA); p-AMPK (Cat# 2535T, RRID: AB_331250, CST, USA); Phospho-sQsTM1/p62 (Ser403) (Cat# F0955, Uniprot ID: Q13501, Selleck, USA); LC3A/B (Cat# F0144, Uniprot ID: Q9H492, Selleck, USA); HRP-conjugated Goat anti-Mouse IgG (H+L) (Cat# AS003, AB _2769851, ABclonal, China); HRP-conjugated Goat anti-Rabbit IgG (H+L) (Cat# AS014, RRID: AB_2769854, ABclonal, China ).

### β-Galactosidase Staining

After light exposure, cells were washed once in PBS and fixed with 1 mL of β-galactosidase fixative solution (Beyotime, Cat# C0602, China) for 15 min at room temperature. The cells were then washed three times with PBS. Subsequently, 1 mL of β-galactosidase staining solution was added to each well, and the plates were incubated overnight at 37 °C in a humidified chamber. Stained cells were then examined and imaged under a bright-field microscope.

### Reactive Oxygen Species (ROS) Detection

For C. elegans, worms were washed with PBS and incubated with 2 μL of 50 μM DCFH-DA (MedChem Express, Cat# HY-D0940, USA) in 100 μL PBS at 20 °C for 6 h in the dark. Fluorescence images were captured using a fluorescence microscope (Olympus, Japan) and quantified using ImageJ software, with fluorescence intensity normalized to worm body area. For mammalian cells, DCFH-DA was diluted 1:1000 in serum-free medium to a final concentration of 10 μM, and cells were incubated at 37 °C in the dark for 0.5-4 h. After washing, fluorescence was imaged and analyzed using ImageJ software, with intensity normalized to cell count or surface area.

### MDA, SOD, and CAT Assays

After light exposure, C. elegans were washed three times with M9 buffer to remove surface bacteria. Nematodes were collected and lysed by ice-cold ultrasonic disruption. Malondialdehyde (MDA) levels, superoxide dismutase (SOD) activity, and catalase (CAT) activity were quantified using commercial assay kits (Nanjing Jiancheng Bioengineering Institute, China) according to the manufacturer’s instructions. Protein concentrations were determined using a bicinchoninic acid (BCA) Protein Assay Kit, and all measurements were normalized to total protein content.

### Cells maintenance

HFF-1 cells (Yizefeng Biotechnology Co., Ltd., Shanghai, China) were cultured in Dulbecco’s Modified Eagle Medium (DMEM) supplemented with 15% fetal bovine serum (FBS). Cells were maintained at 37°C in a humidified incubator with 5% CO_2_. Unless otherwise specified, cells were cultured in phenol red-free medium and exposed to red light (625-635 nm) at 200 lux under open-lid conditions.

### Cell Viability Assessment

Cell viability was assessed using the Cell Counting Kit-8 (CCK-8; abbkine, Cat#: BMU106-CN, China) following the manufacturer’s protocol. HFF-1 cells were seeded in 96-well plates at a density of 1 × 10^4^ cells per well and incubated overnight. After light exposure (with lids removed), 100 μL of DMEM containing 10% CCK-8 solution was added to each well. Plates were incubated at 37 °C for 1 h, and absorbance was measured at 450 nm using a microplate reader. All experiments were conducted in triplicate.

**Figure 1.**
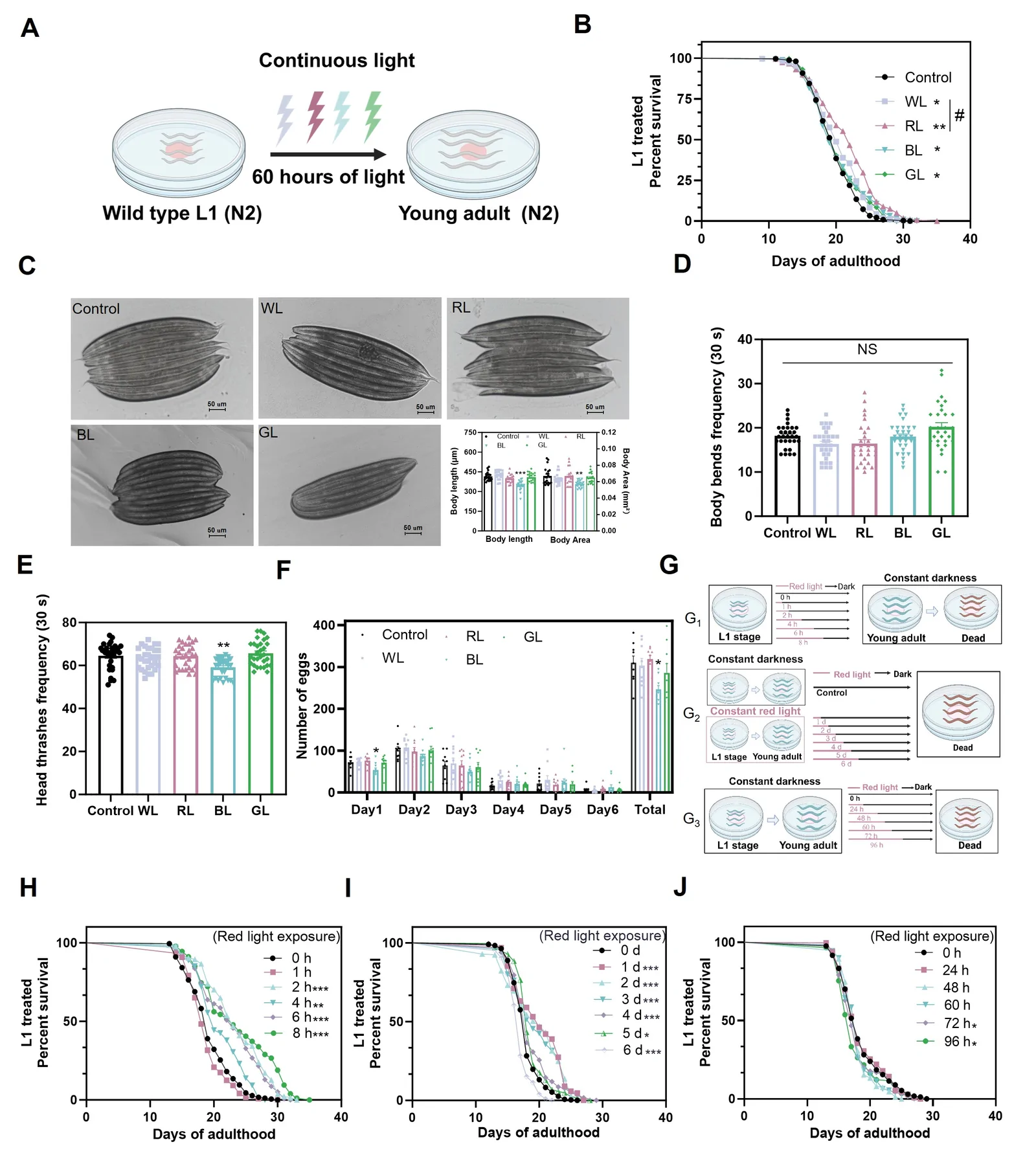
**Early-life exposure to specific light wavelengths modulates lifespan and physiological indices in** *C. elegans.* (A) Experimental setup for exposing L1-stage wild-type (WT) N2 worms to white (380-750 nm), red (630 ± 5 nm), blue (470 ± 5 nm), or green (525 ± 5 nm) light (200 lux) for 60 h. (**B**) Survival of WT worms following early-life red, blue, green, or white light exposure, with light withdrawn prior to the first day of adulthood (n = 328 (control), 301 (white), 299 (red), 279 (blue), 305 (green)). (**C**) Representative images and quantification of body length and surface area at the end of light exposure (n = 20 per group; scale bar, 50 μm). (D, E) Locomotor performance immediately after light exposure: body bend frequency (D) and head thrash frequency (E) (n = 30 per group). (**F**) Daily and cumulative egg production under different light conditions (n = 9 per group). (**G**) Schematic of experimental light exposure schedules. (G_1_), Minimal effective light dose for lifespan extension. (G_2_), Maximal tolerated light dose. (G_3_), Temporal window for longevity promotion. (**H**) Survival of WT worms exposed to red light (0-8 h/day) from L1 to young adult stage, followed by light withdrawal (n = 177 (0 h), 120 (1 h), 134 (2 h), 108 (4 h), 116 (6 h), 180 (8 h)). (**I**) Survival of WT worms continuously exposed to red light from L1 to young adult stage, with additional exposure for 0-6 days in adulthood (n = 304 (0 d), 190 (1 d), 197 (2 d), 101 (3 d), 117 (4 d), 250 (5 d), 106 (6 d)). (**J**) Survival of WT worms exposed to red light for 0-96 h during the young adult stage, followed by light withdrawal (n = 167 (0 h), 245 (24 h), 150 (48 h), 200 (60 h), 248 (72 h), 303 (96 h)). All assays were independently repeated three times. In all panels, “n” denotes the exact number of individual worms per group (biological replicates). All data are mean ± SD. Statistical significance was determined by log-rank (Mantel-Cox) test (B, H-J) or one-way ANOVA with Dunnett’s multiple comparisons test (C-F). NS, not significant; ^*^P < 0.05; ^**^P < 0.01; ^***^P < 0.001 *vs* control, 0 h, or 0 d, as appropriate.

### Selection of representative images

For Western blot analyses, all experiments were independently repeated at least three times under identical conditions. From the resulting replicate blots, one representative image was randomly selected for presentation. For microscopy-based imaging (including fluorescence microscopy, transmission electron microscopy, and conventional light microscopy), images were captured from multiple randomly selected fields of view, and one representative image was randomly chosen for inclusion in the manuscript. All images were acquired using identical imaging parameters within each experiment.

### Statistical analysis

All statistical analyses were conducted using GraphPad Prism version 8.0.2. Data are presented as mean ± standard error of the mean (SEM) or mean ± Standard Deviation (SD). For comparisons between two groups, normally distributed datasets with a sample size of N ≥ 6 were analyzed using an unpaired Student’s *t*-test, whereas datasets with N < 6 or non-normally distributed data were evaluated using the Mann-Whitney U test. Comparisons among more than two groups were performed using one-way analysis of variance (ANOVA) for normally distributed data, followed by Tukey’s post hoc test. Kaplan-Meier survival curves were analyzed using the log-rank (Mantel-Cox) test. *P*<0.05 was considered statistically significant. * *P* < 0.05; ** *P* < 0.01; *** *P* < 0.001; NS, not significant. All data acquisition and statistical analyses were conducted by investigators blinded to group allocation.

Additional details for all methods are provided in Supplementary data.

## RESULTS

### Systematic wavelength screening reveals red light as a longevity-promoting and physiologically safe intervention in *C. elegans*

To investigate the long-term physiological effects of early-life exposure to different light wavelengths, we established a full-spectrum illumination platform (Supplementary Fig. 1). *C.elegans* were exposed to specific wavelengths during critical developmental stages (L1 to young adult), after which light exposure was withdrawn, and worms were maintained in standard dark conditions for lifespan assessment. Survival analysis revealed that all light-exposed groups exhibited significantly extended lifespans compared to the dark control group (19.78 ± 0.18 days). Specifically, the average lifespans were 20.46 ± 0.22 days for white light, 21.66 ± 0.28 days for red light, 20.15 ± 0.24 days for blue light, and 20.19 ± 0.21 days for green light (Fig 1A and B). Among these, red light exposure produced the most substantial extension (+10.34 %), while blue and green light conferred modest benefits.

To further assess the impact of wavelength-specific light exposure on adult *C. elegans* fitness, we evaluated developmental parameters. As shown in (Fig 1C, continuous blue light exposure during early life significantly inhibited growth, resulting in reduced body length and surface area (-14.6 %) compared to dark controls. In contrast, worms exposed to white, red, or green light showed no significant differences in size, indicating that blue light uniquely disrupts *C. elegans* development during early life.

We next assessed post-exposure locomotion and reproductive capacity. As shown in Fig. 1D, body bends frequency remained unchanged across all light conditions when compared with the dark control. Notably, blue light exposure significantly reduced head thrashing frequency by 8.4 % within 30 s (Fig. 1E), while other wavelengths had no effect. Blue light also markedly decreased reproductive output, with a 20.7 % reduction in total brood size relative to dark controls, whereas other wavelengths showed no impact (Fig. 1F). Importantly, hatch rates were unaffected across all light conditions (Supplementary Fig. 2A, B). Collectively, these findings suggest that early-life red light exposure represents a promising non-pharmacological strategy for extending lifespan. Accordingly, we selected red light for further mechanistic exploration.

**Figure 2.**
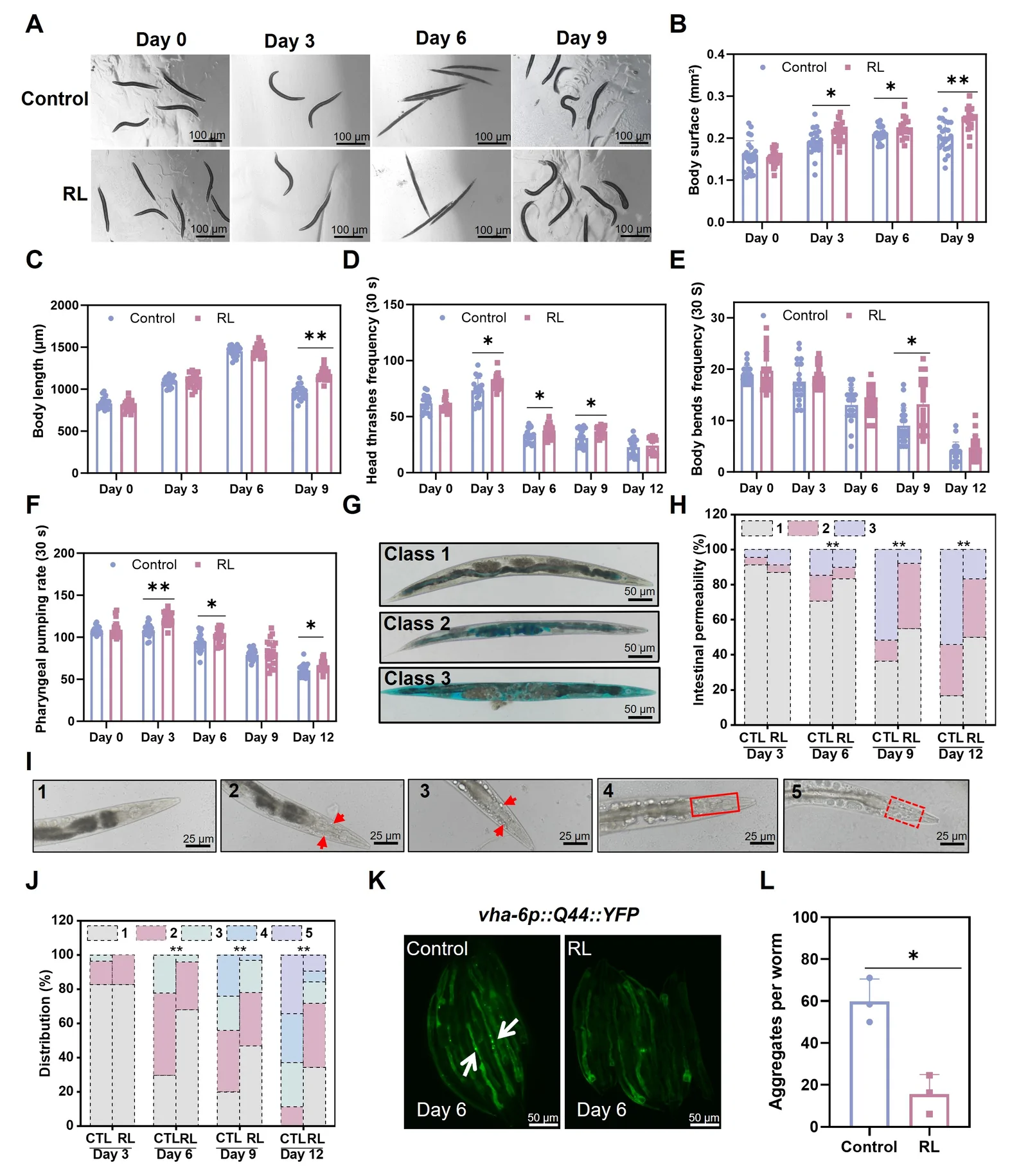
**Early adulthood red light exposure improves somatic health and protein homeostasis in** *C. elegans*. (**A-C**) Representative images (A) and quantification of body surface area (B) and body length (C) in adult WT worms at days 0, 3, 6, and 9 after red light exposure (n = control: 24 (day 0), 21 (day 3), 25 (day 6), 23 (day 9); RL: 28 (day 0), 24 (day 3), 20 (day 6), 25 (day 9); scale bar, 100 μm). (**D-F**) Quantification of locomotor and feeding performance at days 0, 3, 6, 9, and 12 after red light exposure: head thrash frequency (D), body bend frequency (E), and pharyngeal pumping rate (F) (n = 20 per group). (**G-H**), Body-cavity barrier integrity assessed via dye leakage assay at days 3, 6, 9, and 12 (n = control: 23 (day 3), 34 (day 6), 33 (day 9), 24 (day 12); RL: 23 (day 3), 30 (day 6), 51 (day 9), 30 (day 12); scale bar, 50 μm). (I, J) Age-dependent pharyngeal deterioration assessed at days 3, 6, 9, and 12. (**I**) Representative bright-field images illustrating the 5-class scale: Class 1 (young/intact), Class 2 (mild), Class 3 (moderate), Class 4 (marked), Class 5 (severe). Red arrows indicate vesicle-like vacuoles; solid-line boxes show muscle discontinuity and lumen collapse; dashed boxes highlight fully degenerated structures. (**J**), Quantification of deterioration scores (n = control: 29 (day 3), 27 (day 6), 25 (day 9), 35 (day 12); RL: 29 (day 3), 25 (day 6), 32 (day 9), 32 (day 12); scale bar, 25 μm). (K, L) Red light reduced age-associated protein aggregation, visualized by vha-6p::Q44::YFP expression in intestinal cells. White arrows indicate puncta (n = control: 30; RL: 31; scale bar, 50 μm). All assays were independently repeated three times. In all panels, “n” denotes the exact number of individual worms per group (biological replicates). Data are mean ± SEM for panel (G) and mean ± SD for all others. Statistical significance was determined by two-tailed unpaired Student’s *t*-test. NS, not significant; ^*^P < 0.05; ^**^P < 0.01 *vs* Control.

To determine the temporal requirements for red light-mediated longevity, synchronized L1 larvae were exposed to increasing daily photoperiods (1 h, 2 h, 4 h, 6 h, and 8 h) until reaching young adulthood. Survival analysis revealed that only 2 hours (h) of daily exposure extended lifespan by 12.13%. Notably, extending the photoperiod beyond 2 h did not provide additional benefits, suggesting a saturation effect (Fig. 1G1, H).

To further define the effective duration of red-light exposure, synchronized L1 larvae were exposed for varying durations (1-6 days) during their development from larval to young adult stages. Our results showed that extending red light exposure beyond 5 days into adulthood not only eliminated longevity benefits but also induced detrimental effects (Fig. 1G2, I). These findings suggest a dose-dependent effect of red light, where exceeding the optimal threshold results in adverse outcomes.

To assess whether the longevity benefits of red light are specific to certain developmental stages, synchronized *C. elegans* at the young adult stage were exposed to red light for varying durations (24-96 h) under the same illumination parameters (Fig. 1G3, J). Strikingly, none of the adult-exposed groups showed lifespan extension when compared with the dark control. This contrasts sharply with the 1 0.34 % lifespan extension observed in L1-stage worms exposed to the same cumulative energy (60 h), highlighting the necessity of an early developmental window (L1 to young adult) for realizing red light-mediated longevity benefits.

To rule out confounding variables, we confirmed that the minor increase in agar surface temperature (~0.6 °C) during red light exposure had no effect on lifespan (Supplementary Fig. 3 and Fig. 4A). Moreover, lifespan extension was still observed when worms were fed heat-killed (HK) *Escherichia coli* (E. coli), although the magnitude was modestly reduced when compared with live bacteria (Supplementary Fig. 4C). This indicates that red light exerts a direct pro-longevity effect on *C. elegans*, while the presence of metabolically active bacteria may further enhance this benefit. Together, these findings demonstrate that early-life red light exposure promotes longevity primarily via a direct effect on the host, with a possible contribution from live bacterial interactions.

### Red light exposure during early-life stage improves multiple physiological healthspan indicators in *C. elegans*

Early-life red light exposure not only extended lifespan but also mitigated age-associated decline in adulthood. As shown in Fig. 2A-C, red light treatment increased body length and surface area, with significant differences in surface area observed by day 3 of adulthood and in body length by day 9. The observed increase in surface area may be associated with elevated levels of cytoskeletal proteins [23]. To test this hypothesis, we measured β-actin and β-tubulin expression in day 6 adults, using GAPDH as a loading control (Supplementary Fig. 5A-C). No significant differences were found between red light-treated and control groups, suggesting that red light supports somatic maintenance without inducing cytoskeletal overexpression.

Red-light exposure mitigates age-associated neuromuscular degeneration in nematodes, as evidenced by head swing frequency, body bending rate, and pharyngeal pumping rate in the red light-treated group, all of which remained superior to those of the dark control group at equivalent age stages (Fig. 2D-F). It also mitigated the decline in intestinal barrier integrity (Fig. 2G, H) and delayed pharyngeal deterioration (Fig. 2I, J), although lipofuscin accumulation remained unchanged (Supplementary Fig. 5D). As polyglutamine (polyQ) protein aggregation is a hallmark of aging and commonly observed in aged neurons[24], we next examined whether red light affects age-associated proteostasis decline. Analysis of Q44::YFP aggregation in day 6 adults revealed a significant reduction in polyQ puncta in the red light-treated group (Fig. 2K, L), indicating that proteostasis deterioration was attenuated relative to the dark control group. Collectively, these findings suggest that early-life red light exposure promotes healthspan by delaying the decline of somatic function and maintaining proteostasis.

### Early life red light exposure triggers functional MQC reprogramming to enhance mitochondrial function across lifespan

Mitochondrial dysfunction is a hallmark of aging [28]. To investigate whether early-life red light exposure modulates mitochondrial activity, we performed Seahorse metabolic flux analysis immediately after light withdrawal (60 h) and again six days later. Exposure from the L1 to young adult stage led to a 41.6 % reduction in basal oxygen consumption rate (OCR) and a 27.4 % decrease in spare respiratory capacity (Fig. 3A, B; *P* = 0.0653 and 0.1349, respectively). Six days after cessation of red light, basal OCR was 34.0% higher than in dark controls (Fig. 3C; *P* = 0.0980), whereas spare respiratory capacity was significantly increased by 41.0 % (Fig. 3D). ATP-linked respiration remained unchanged (Supplementary Fig. 6A, B). Collectively, these data suggest that early-life red light exposure induces a transient, non-significant suppression of mitochondrial activity. In line with these observations, ATP levels in nematodes were significantly reduced by 24.7 % after 60 h of red-light exposure, indicating an acute suppression of mitochondrial energy output. By day 6 post-exposure, ATP levels had rebounded to exceed those of the control group (Fig. 3E), suggesting a gradual recovery of mitochondrial function.

**Figure 3.**
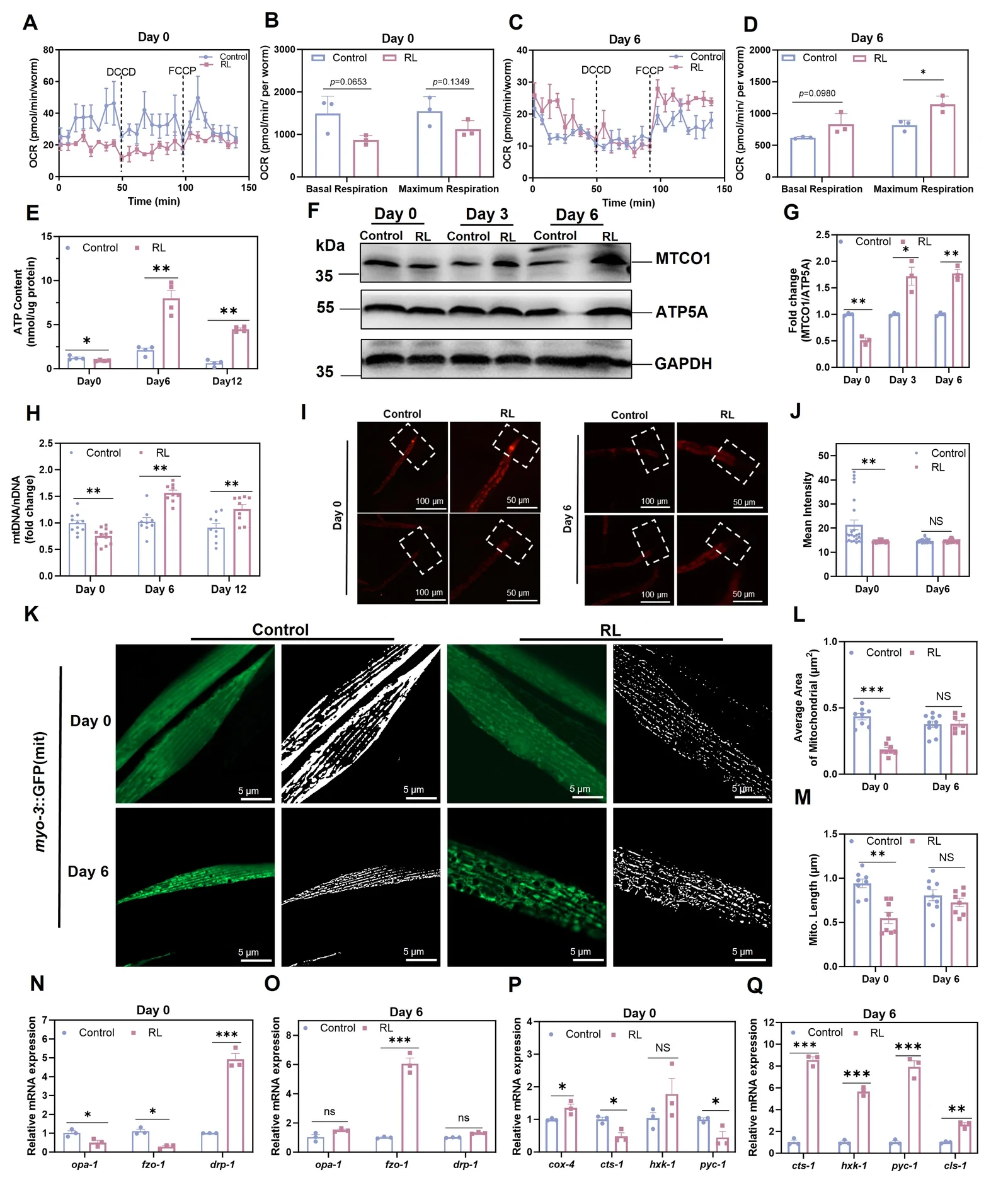
**Early-life red light exposure transiently modifies mitochondrial homeostasis and promotes functional remodeling in later life**. (**A-D**) Whole-worm oxygen consumption rate (OCR) analysis at day 0 and day 6 after red light exposure. Basal OCR was measured, followed by sequential injection of DCCD and FCCP to determine maximal respiration (FCCP-stimulated) and spare respiratory capacity (maximal minus basal OCR). Values were normalized to worm number per well (n = control: 17, 19, 20 (day 0), 20, 19, 15 (day 6); RL: 18, 17, 16 (day 0), 21, 21, 16 (day 6); numbers indicate worms counted per biological replicate, three replicates in total). (**E**), Whole-body ATP levels at days 0, 6, and 12 after red light exposure. (F, G) Western blot analysis (F) of OXPHOS complex subunits encoded by nuclear (ATP5A) and mitochondrial (MTCO1) DNA at days 0, 3, and 6 after red light exposure, with densitometric quantification (G) (n=3 per group). (**H**), Relative mitochondrial DNA content (nd-1/act-3) compared with control worms at each time point (n = control: 11 (day 0), 9 (day 6), 9 (day 12); RL: 12 (day 0), 9 (day 6), 9 (day 12)). (I, J), Representative fluorescence images (I) and quantification (J) of TMRE staining at days 0 and 6 (n = control: 24 (day 0), 23 (day 6); RL: 25 (day 0), 27 (day 6); scale bar, 50 μm). (**K**) Mitochondrial morphology in body wall muscle cells of pmyo-3::mito::GFP worms at days 0 and 6. The left panels show raw GFP images, and right panels display the corresponding binarized/outline masks that were generated for quantitative analysis of mitochondrial length and average area (scale bar, 5 μm). (L, M) Quantification of mitochondrial area (L) and length (M) from panel K (n = control: 8 (day 0), 10 (day 6); RL: 8 (day 0), 7 (day 6)). (N, O) Relative mRNA expression of mitochondrial fusion and fission genes compared with control worms at days 0 and 6. (P, Q) Relative mRNA expression of metabolic genes compared with control worms at days 0 and 6. All assays were independently repeated three times. “n” indicates the exact number of worms per group, except in panel (G), where n = 3 denotes three independent biological samples for Western blot quantification. Data are mean ± SD (A-D, J, L, M) or mean ± SEM (E, G, H, N-Q). Statistical significance was determined by two-tailed unpaired Student’s *t*-test unless otherwise indicated. NS, not significant; ^*^P < 0.05; ^**^P < 0.01; ^***^ *P*< 0.001 *vs* Control.

To assess mitochondrial-nuclear coordination, we quantified the ratio of mitochondrial-encoded MTCO1 to nuclear-encoded ATP5A. This ratio was reduced in red light-exposed worms. However, following light withdrawal, the ratio gradually recovered and by day 3 post-exposure exceeded that of dark controls (Fig. 3F, G), indicating a transient stoichiometric imbalance between mitochondrial and nuclear components. Moreover, early-life red light exposure reduced the mitochondrial DNA (mtDNA) to nuclear DNA (nDNA) ratio and mitochondrial gene expression, which were modestly decreased after exposure but fully restored by day 6 (Fig. 3H; Supplementary Fig. 7A, B), suggesting that early-life red light exposure disrupts mitochondrial biogenesis. Similarly, mitochondrial membrane potential was significantly reduced immediately after light withdrawal but returned to baseline levels by day 6 (Fig. 3I, J), suggesting restoration of mitochondrial electrochemical integrity. Given the critical role of mitochondrial dynamics in regulating organelle morphology and function [29], we examined mitochondrial structure using *C. elegans* expressing mitochondrial-targeted GFP (pmyo-3::mito::GFP). Red light exposure promoted mitochondrial fragmentation, indicative of increased fission. However, by day 6 post-light withdrawal, mitochondrial networks had returned to baseline morphology (Fig. 3K), with mean mitochondrial area and length indistinguishable from dark controls (Fig. 3L, M), indicating that mitochondria had adapted to red light-induced stress. At the transcriptional level, red light downregulated the expression of fusion-related genes *fzo-1* and *opa-1*, while upregulating the fission gene *drp-1*. Notably, *fzo-1* expression was significantly elevated by day 6 after light removal (Fig. 3K, L), indicating a compensatory restoration of mitochondrial fusion capacity.

To further assess metabolic adaptation, we analyzed the expression of key metabolic enzymes genes. Following red light exposure, expression of citrate synthase-1 (*cts-1*) and pyruvate carboxylase (*pyc-1*) was downregulated; these genes encode citrate synthase and pyruvate carboxylase, which participate in the tricarboxylic acid (TCA) cycle and gluconeogenesis, respectively. Expression of hexokinase (*hxk-1*) remained unchanged, whereas cytochrome c oxidase subunit IV (*cox-4*) was modestly upregulated (Fig. 3P). By day 6 following light withdrawal, *cts-1*, *hxk-1*, and *pyc-1* were significantly upregulated. In addition, we measured expression of the cardiolipin synthase gene (*cls-1)*, which encodes the enzyme catalyzing cardiolipin biosynthesis and contributes to maintenance of the mitochondrial inner membrane and the stability of respiratory-chain supercomplexes [30]; its mRNA level was also significantly increased at day 6 (Fig. 3Q), in parallel with the recovery of spare respiratory capacity. Taken together, these results are consistent with an initial, transient perturbation of mitochondrial homeostasis after early-life red light exposure, followed by adaptive remodeling of the mitochondrial inner membrane and metabolic function.

### MQC-mediated proteostasis: early-life red light activates UPR^MT^ for mitochondrial stress adaptation and lifespan extension

Given the long-lasting metabolic effects of early-life red light exposure, we next investigated its potential to activate the UPR^MT^, a stress pathway that maintains mitochondrial proteostasis and coordinates cellular adaptation [31]. We first assessed the transcriptional activation of canonical markers of UPR^MT^, Endoplasmic reticulum unfolded protein response (UPR^ER^), and cytosolic unfolded protein response (UPR^CT^) at day 0 (immediately after completion of the early-life red-light exposure).

**Figure 4.**
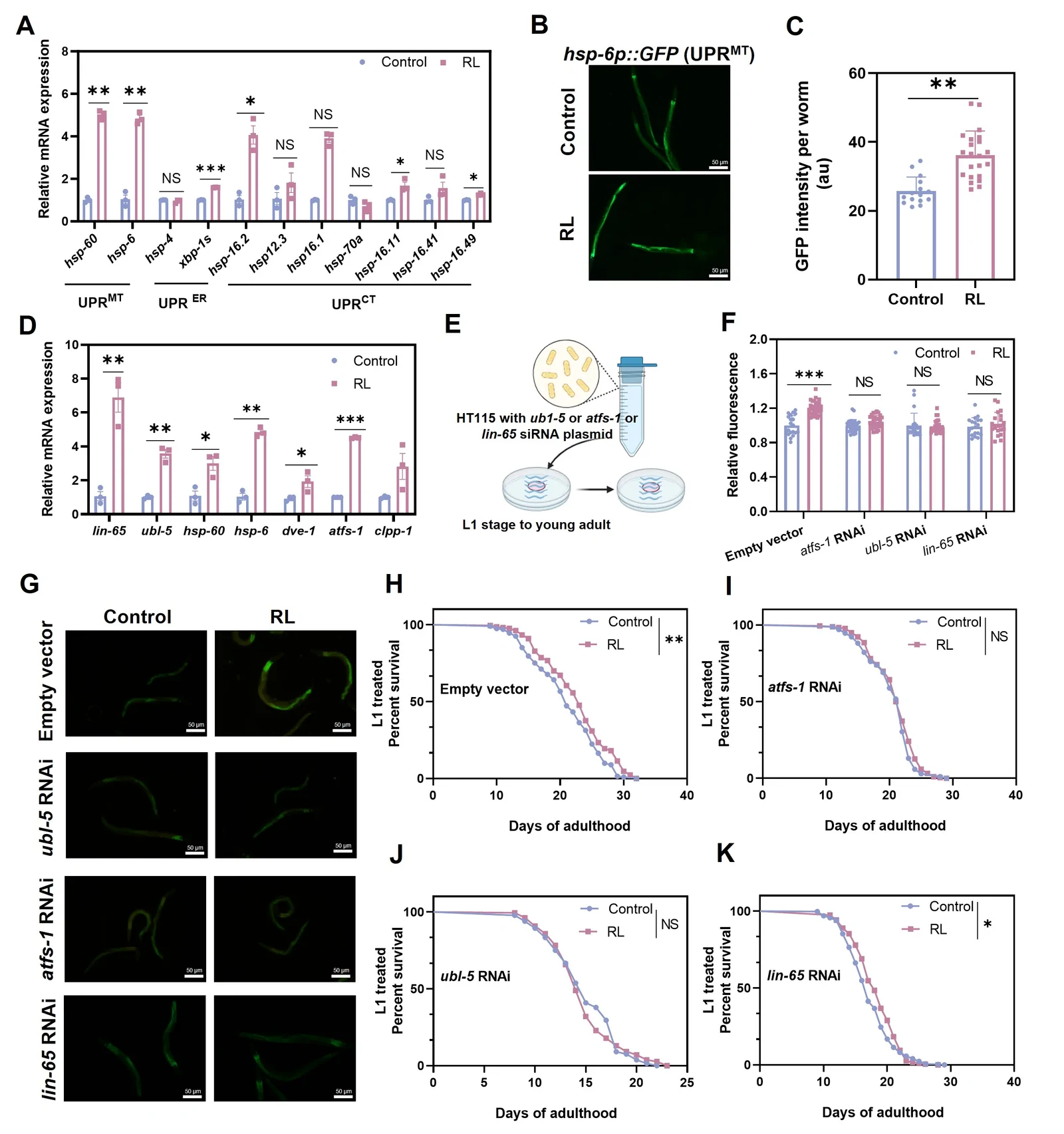
**Red light activates the mitochondrial unfolded protein response (UPR^MT^) in** *C. elegans*. (**A**) Relative mRNA expression levels of canonical UPR markers compared with control worms at day 0 post exposure. (B, C) Representative fluorescence images (B) and quantification (C) of Phsp-6::GFP at day 0 post exposure (n = control: 15; RL: 22; scale bar, 50 μm). (**D**) Relative mRNA expression of core UPR^MT^ genes compared with control worms at day 0 post exposure. (**E**) Schematic of RNAi-based knockdown strategy targeting *atfs-1*, *ubl-5*, and *lin-65* to assess their role in UPR^MT^ activation. (F, G) Representative images (F) and quantification (G) of *Phsp-6::GFP* fluorescence in worms at day 0 post exposure under RNAi or control conditions, relative fluorescence intensity compared with worms fed empty vector (L4440) bacteria (n = control: 20 (Empty vector), 30 (*atfs-1* RNAi), 22 (*ubl-5* RNAi), 19 (*lin-65* RNAi); RL: 30 (Empty vector), 29 (*atfs-1* RNAi), 29 (*ubl-5* RNAi), 20 (*lin-65* RNAi); scale bar, 50 μm). (**H-K**), Survival of worms subjected to early-life red-light exposure followed by maintenance in darkness; lifespan monitoring started at day 0 (n = control: 201 (Empty vector), 168 (*atfs-1* RNAi), 132 (*ubl-5* RNAi), 297 (*lin-65* RNAi); RL: 210 (Empty vector), 182 (*atfs-1* RNAi), 184 (*ubl-5* RNAi), 190 (*lin-65* RNAi)). For panels E-K, worms were fed from hatching with either control bacteria (empty vector, L4440) or bacteria expressing dsRNA targeting *atfs-1*, *ubl-5*, or *lin-65*. For all other panels, worms were fed *E. coli* OP50. All assays were independently repeated three times. “n” indicates the exact number of individual worms per group (biological replicates). Data are mean ± SD (C, F, H-K) or mean ± SEM (A, D). Statistical significance was determined by two-tailed unpaired Student’s *t*-test (A, C, D, F) or log-rank (Mantel-Cox) test (H-K). NS, not significant; ^*^P < 0.05; ^**^P < 0.01; ^***^P < 0.001 *vs* Control.

**Figure 5.**
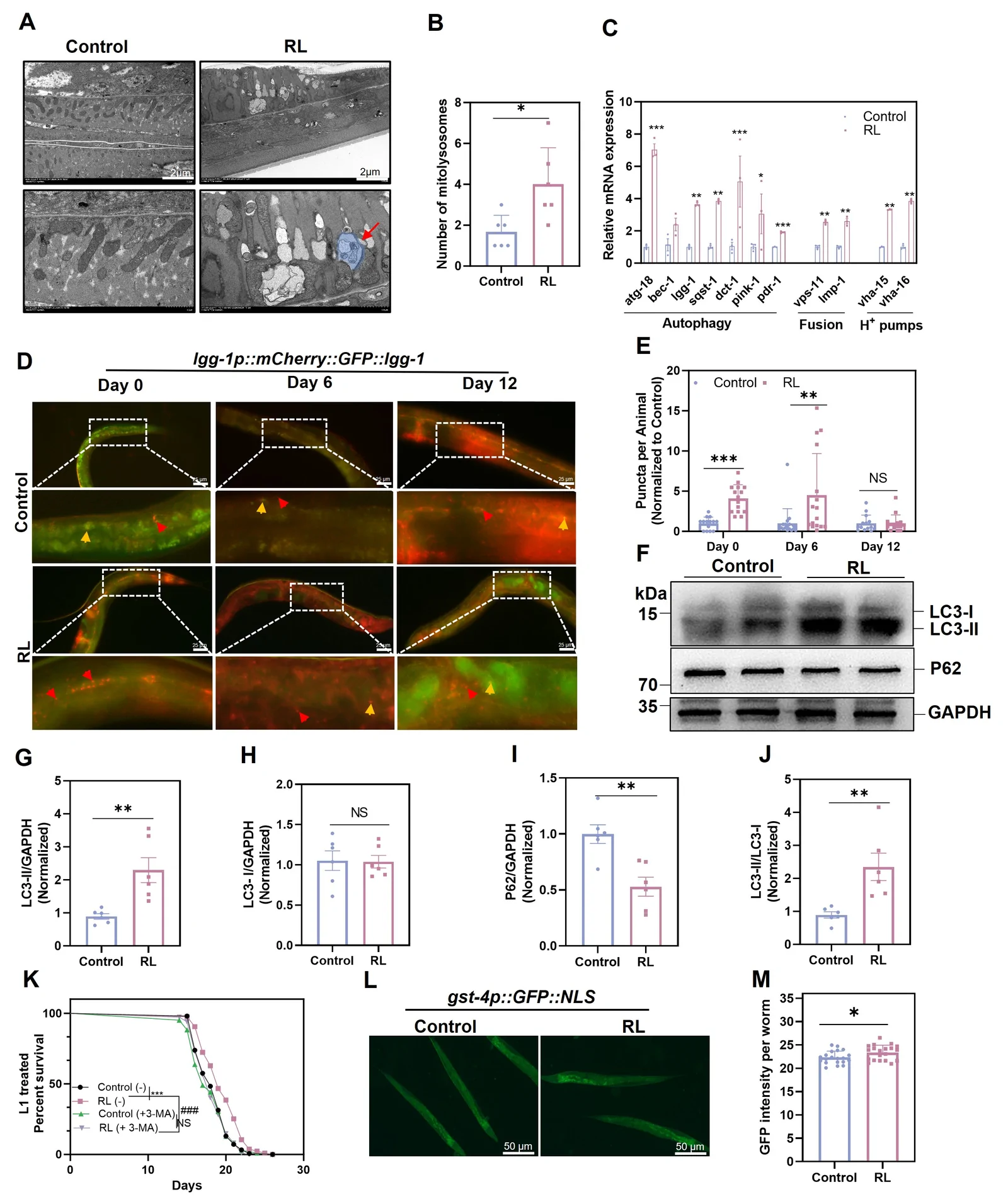
**Red light exposure activates mitochondrial quality control through mitophagy in** *C. elegans*. (**A**) Representative TEM images of mitochondria in WT worms after red light exposure. Normal mitochondria appear round, oval, or crescent-shaped with intact cristae, while aged mitochondria show swelling, vacuolization, or cristae disruption. Red light-treated worms show an increase in mitolysosomes (blue-filled areas indicated by red arrows; Scale bars: 2 μm (top), 1 μm (bottom). (**B**) Quantification of mitolysosomes from six independent TEM micrographs (n=6 per group). (**C**) Relative mRNA expression of autophagy-related genes compared with control in response to red light exposure. (D, E) Representative fluorescence images (D) and quantification (E) of autophagic structures in MAH215 worms after red light exposure. Autophagosomes (Aps, mCherry/GFP double-positive, yellow arrowheads) and autolysosomes (Als, mCherry-only, red arrowheads) are indicated; quantification is based on the number of autolysosomes per worm (n = control: 14 (day 0), 19 (day 6), 14 (day 12); RL: 15 (day 0), 16 (day 6), 15 (day 12); scale bar, 25 μm). (**F-J**), Western blot analysis of LC3-I, LC3-II, and P62 expression after red light exposure (F), with densitometric quantification in G-J (n=6 per group). (**K**), Survival of WT (N2) worms exposed to red light with or without the autophagy inhibitor 3-methyladenine (3-MA) during early life (n = control (-): 153, RL (-): 210, control (+3-MA): 102, RL (+3-MA): 186). (L, M) Representative fluorescence images (L) and quantification (M) of *gst-4p::GFP* signal after red light exposure (n = 20 per group; scale bar, 50 μm). All assays were independently repeated three times. In panels (E, M), “n” indicates the exact number of individual worms per group (biological replicates); in panel (B), where n = 6 denotes six independent TEM micrographs used for mitolysosome quantification, and in panels (G-J), where n = 6 denotes six independent biological samples for Western blot quantification. Data are mean ± SD (B, E, M) or mean ± SEM (C, G-K). All assays were independently repeated three times. Statistical significance was determined by two-tailed unpaired Student’s *t*-test (A-J, M) or log-rank (Mantel-Cox) test (K). NS, not significant; ^*^P < 0.05; ^**^P < 0.01 *vs* Control.

Notably, red light significantly upregulated the expression of *hsp-6* and *hsp-60*, surpassing UPR^CT^ pathway markers (Fig. 4A). To validate UPR^MT^ activation *in vivo*, we used transgenic *C. elegans* expressing GFP reporters driven specifically by the *hsp-6* promoter, a canonical marker of UPR^MT^. Additionally, we employed reporters for the UPR^CT^ (Phsp-16.2::GFP) and UPR^ER^ (Phsp-4::GFP) to assess the activation of these additional stress pathways. Consistently, at day 0, *Phsp-6::GFP* fluorescence intensity was elevated 1.4-fold in red light-treated worms compared to controls (Fig. 4B, C), while Phsp-16.2::GFP fluorescence increased by 1.14-fold (Supplementary Fig. 8A, B). No significant change in Phsp-4::GFP fluorescence was observed, indicating that the endoplasmic reticulum stress response was not markedly induced (Supplementary Fig. 8C, D), consistent with the results from endogenous transcriptional analysis. To further investigate the specificity of UPR^MT^ activation, we assessed the transcript levels at day 0 of key regulators, including the transcription factor activating transcription factor-1 (*atfs-1*), the co-regulators dorsal-ventral embryo-1 (*dve-1*) and ubiquitin-like protein 5 (*ubl-5*), the mitochondrial protease CLPP-like protease 1 (*clpp-1*), and the H3K9 Methyltransferase Lamin-Interacting Protein 65 (*lin-65*). Red light significantly upregulated *atfs-1*, *ubl-5*, and *lin-65* expression (Fig. 4D), all of which are critical for the induction and maintenance of UPR^MT^. To assess the temporal dynamics of this response, we next quantified *hsp-6::GFP* fluorescence at day 0 (immediately after light withdrawal), day 6, and day 12. *Phsp-6::GFP* levels remained significantly elevated in red light-treated worms relative to dark controls at all examined time points (Supplementary Fig. 9A, B), indicating a sustained activation of UPR^MT^ rather than a transient induction. Importantly, a mild temperature increase of 0.6 °C alone did not induce *hsp-6::GFP* activation (Supplementary Fig. 9C, D), further excluding thermal effects as a confounding factor. This persistent signal temporally coincided with the recovery of mitochondrial respiratory capacity and the upregulation of metabolic and membrane-remodeling genes described above, suggesting that prolonged UPR^MT^ activity may contribute to the maintenance of mitochondrial homeostasis and the longevity benefits conferred by early-life red light exposure.

To establish the functional relevance of this sustained UPR^MT^ activation, we performed RNAi-mediated knockdown of *atfs-1*, *ubl-5*, and *lin-65* (silencing efficiency > 50 %; Fig. 4E; Supplementary Fig. 10A-C). The red light-induced increase in Phsp-6::GFP fluorescence at day 0 was abolished in all three knockdown conditions (Fig. 4F, G), indicating that red light activates UPR^MT^ through canonical signaling components. Moreover, lifespan assays showed that red light failed to extend lifespan in *ubl-5* or *atfs-1* knockdown worms (Fig. 4H-J), whereas *lin-65* knockdown attenuated the longevity benefit without abolishing it (Fig. 4K). Collectively, these data demonstrate that early-life red light exposure induces UPR^MT^ activation through classical regulatory factors and persists after light removal; this sustained activation is essential for the extended lifespan of *C. elegans*.

### MQC-mitophagy enhancement via early-life red light exposure improved longevity and stress resistance

While the UPR^MT^ response provides an immediate defense against mitochondrial stress, its sustained efficacy may require cooperation with additional mitochondrial quality-control mechanisms [32]. Given the persistent metabolic remodeling observed after red light exposure, we hypothesized that mitophagy might also be activated. Transmission electron microscopy (TEM) revealed a marked accumulation of autophagosomes surrounding mitochondria in the body wall muscle cells of red light-treated nematodes (Fig. 5A, B), providing direct morphological evidence for mitophagy activation. These ultrastructural features suggest that early-life red light exposure promotes the removal of damaged or excess mitochondria, thereby contributing to mitochondrial quality control during early development and potentially supporting long-term organismal health.

Although autophagy is primarily regulated through post-translational modifications [33], recent studies have also highlighted the critical role of transcriptional regulation in maintaining cellular homeostasis [34]. To investigate whether early-life red light exposure influences autophagy at the transcriptional level, we quantified the expression of key autophagy-related genes involved in autophagosome biogenesis, autophagosome-lysosome fusion, and lysosomal degradation. Red light exposure significantly upregulated the expression of several autophagy-related genes. Notably, expression of *vps-11* and *imp-1*, which mediate autophagosome-lysosome fusion, was elevated, suggesting that red light enhances this fusion process and accelerates autophagic substrate turnover. Additionally, nematodes exposed to red light showed increased expression of *vha-15* and *vha-16*, key regulators of lysosomal function, supporting the hypothesis that red light modulates lysosomal activity and improves degradative efficiency during autophagy (Fig. 5C).

**Figure 6.**
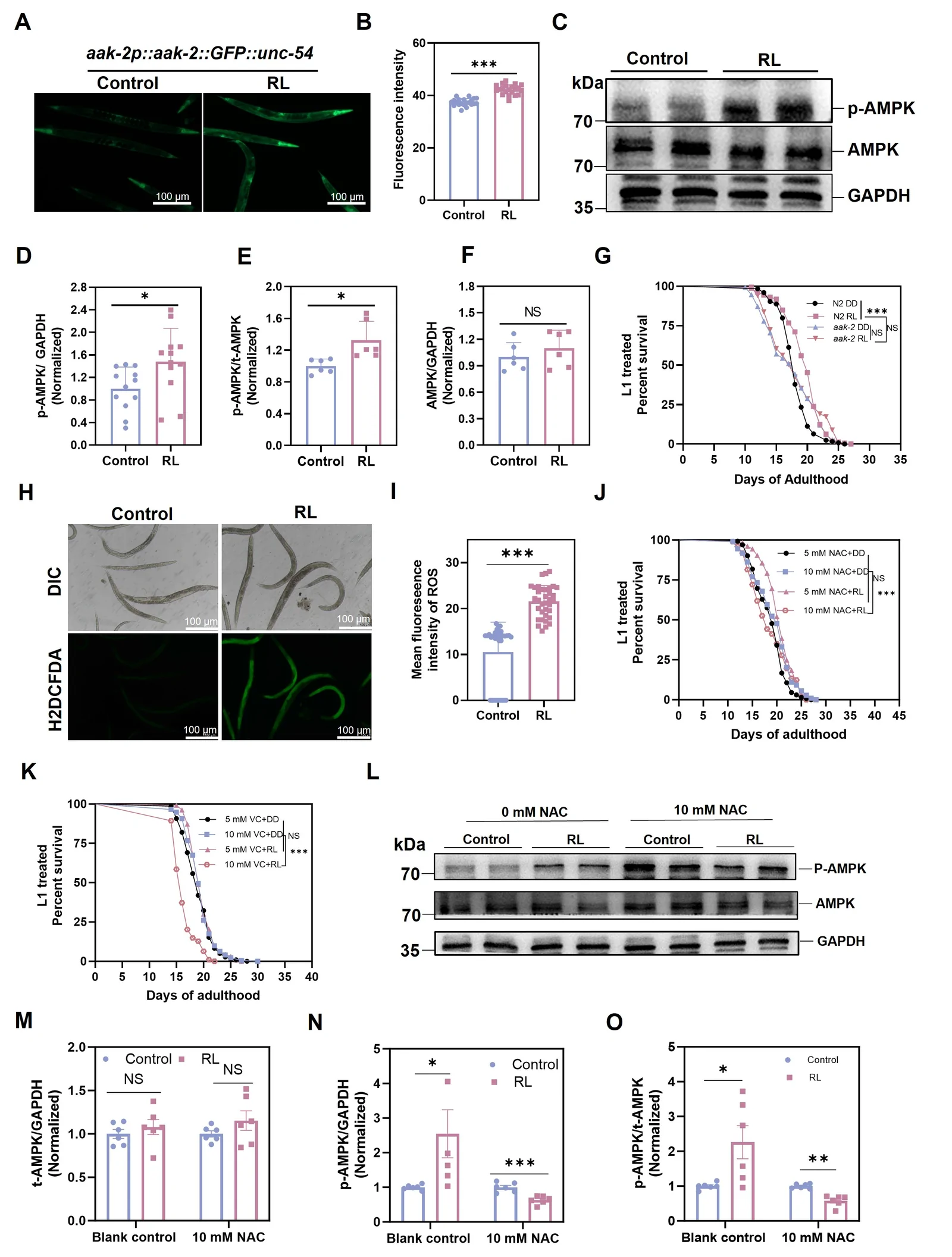
**Red light promotes longevity through a ROS-AMPK signaling axis**. (A, B) Representative fluorescence images (A) and quantification (B) of *aak-2p::aak-2::GFP* expression in WBM60 worms after red light exposure (n = 21 per group; Scale bar, 100 μm). (**C-F**) Western blot analysis of total AMPK and phosphorylated AMPK (p-AMPK) after red light exposure (C), with densitometric quantification in D-F (n=6 per group). (**G**) Survival of AMPK-deficient aak-2(ok524) mutants exposed to red light from the L1 to young adult stage, followed by light withdrawal (n = control: 124 (N2 DD), 149 (aak-2 DD); RL: 159 (N2 RL), 167 (aak-2 RL)). (H, I), Representative images (H) and quantification (L) of whole-body ROS levels measured using the DCFH-DA probe after red light exposure (n = control: 34; RL: 35; Scale bar, 100 μm). (J, K), Survival of WT worms co-treated with red light and the antioxidants NAC (J) or vitamin C (K) at 5 mM and 10 mM during early life. Continuous darkness (DD), (n = control: 132 (5mM NAC+DD), 109 (10mM NAC+DD); RL: 125 (5mM NAC+RL), 97 (10mM NAC+RL)). (**L-O**) Western blot analysis of total AMPK and phosphorylated AMPK (p-AMPK) after red light exposure with or without NAC co-treatment (L), with densitometric quantification in M-O (n=6 per group). All assays were independently repeated three times. “n” indicates the exact number of individual worms per group (biological replicates), except in panels (C-F) and (L-O), where n = 6 denotes six independent biological samples for Western blot quantification. Data are mean ± SD (B, I), all others are mean ± SEM. Statistical significance was determined by two-tailed unpaired Student’s *t*-test (panels B, D-F, I, M-O) or log-rank (Mantel-Cox) test for survival data (panels J, K). NS, not significant; ^*^P < 0.05; ^**^P < 0.01; ^***^P < 0.001 *vs* Control.

To directly monitor autophagic flux in vivo, we employed the MAH215 transgenic strain expressing the tandem-tagged mCherry::GFP::LGG-1 reporter, which allows discrimination of early autophagosomes from late autolysosomes. Red light-treated worms displayed a significant increase in red-only puncta at day 0 and day 6, indicating that early-life red light exposure enhances autophagic flux (Fig. 5D, E). By day 12, however, puncta levels in the red-light group no longer exceeded those in the dark control. Instead, control animals exhibited an age-associated accumulation of autophagic puncta, consistent with Chang *et* al.[35], who reported that autophagosomes and autolysosomes expand with age in a tissue-specific manner, while declining in certain tissues such as neurons. These findings indicate that red light induces a transient activation of autophagy, whereas puncta accumulation in aged controls likely reflects reduced clearance with aging rather than sustained autophagic activity.

In addition, red light exposure induced a marked increase in ATG-18 fluorescence intensity, a core regulator of autophagosome formation (Supplementary Fig.11 A, B). Protein analysis further corroborated these imaging results: red light exposure increased LC3-II levels by 2.6-fold and reduced p62/SQSTM1 by 47 % (Fig. 5F-J), providing robust molecular evidence for mitophagy activation. Functionally, pharmacological inhibition of autophagy with 3-methyladenine (3-MA) during early-life exposure completely abolished the lifespan extension normally conferred by red light (Fig. 5K), demonstrating that autophagy/mitophagy activation is indispensable for the pro-longevity effect.

To investigate upstream regulatory mechanisms, we tested nuclear translocation of canonical transcription factors, including DAF-16/FOXO, SKN-1/NRF2, and HLH-30/TFEB. Surprisingly, none of these factors exhibited detectable nuclear localization upon early-life red light exposure (Supplementary Fig. 12A-D). However, we observed that red light modestly yet significantly upregulated gst-4p::GFP, a well-established transcriptional target of SKN-1 (Fig. 5L, M), suggesting that SKN-1 signaling may be engaged through a non-canonical activation route.

### Red light activates a ROS-dependent AMPK pathway essential for lifespan extension

AMPK is a key regulator of MQC and organismal longevity, integrating energy stress signals through phosphorylation-dependent cascades [36]. To test whether early-life red light exposure activates AMPK signaling, we used a transgenic reporter strain expressing endogenous aak-2p::aak-2::GFP, which allows *in vivo* monitoring of AMPK activation via GFP fluorescence. Red light exposure led to a 12.9 % increase in GFP fluorescence intensity in the aak-2p::aak-2::GFP reporter strain (Fig. 6A, B), indicating transcriptional upregulation of *aak-2*. In parallel, phosphorylated AMPK (p-AMPK) protein levels increased 1.5-fold (Fig. 6C-F). To determine the functional requirement of AMPK activation in the longevity phenotype, we assessed aak-2(ok524) mutants, which lack functional AMPK. Red light failed to extend lifespan in these mutants, with a median lifespan of 18.5 days for controls and 18.7 days for red light-treated animals (Fig. 6G), demonstrating that AMPK activation is essential for the pro-longevity effects of red-light exposure.

Furthermore, to determine whether AMPK is necessary for the transcriptional activation of autophagy and mitochondrial quality-control (MQC) pathways, we measured the mRNA levels of representative genes involved in mitophagy, UPR^MT^, and mitochondrial dynamics in *aak-2(ok524)* mutants following early-life red-light exposure. As shown in Figs 3-5 of the main text, these genes are robustly upregulated by red light in wild-type animals. In contrast, this transcriptional induction was largely abolished in the *aak-2* mutant background (Supplementary Fig. 13A-C), i indicating that AMPK is essential for activating key autophagy- and MQC-related genes at the transcriptional level, and acts as a pivotal signaling node linking red light perception to downstream mitochondrial maintenance programs.

**Figure 7.**
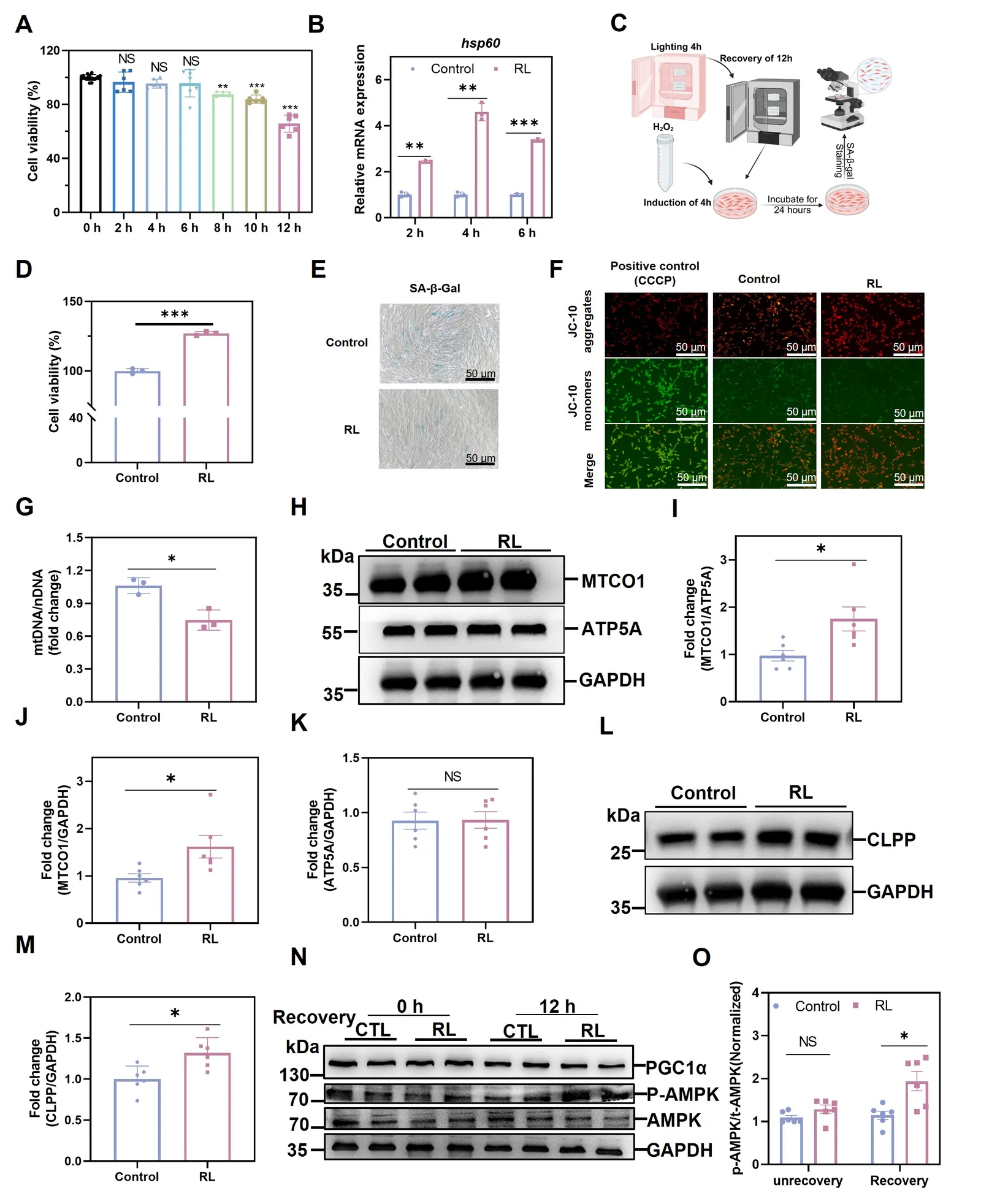
**Red light exposure activates mitochondrial quality control in human fibroblasts**. (**A**) Viability of human dermal fibroblasts (HFF-1) after different durations of red-light exposure, assessed by CCK-8 assay. (**B**) Relative expression of *HSP60* mRNA compared with dark control after red light exposure. (**C**) Schematic of the experimental design showing red light exposure, recovery period, and H__2__O__2__-induced senescence paradigm. (D, E) Cell viability (D) and senescence (E, SA-β-Gal staining) in HFF-1 cells preconditioned with red light (4 h), followed by 12 h recovery and 4 h H_2_O_2_ challenge (Scale bar, 50 μm). (**F**) Representative fluorescence images of mitochondrial membrane potential measured by JC-10 staining after red light exposure. (**G**) Quantification of the mitochondrial-to-nuclear DNA ratio (mtDNA/nDNA) in HFF-1 cells after 4 h of red-light exposure. (**H-K**) Western blot analysis of MTCO1 and ATP5A expression after red light exposure (H), with densitometric quantification in I-K (n=6 per group). (L, M), Western blot analysis of CLPP expression after red light exposure (L), with densitometric quantification in M (n=6 per group). (N, O) Western blot analysis of AMPKα, phospho-AMPKα (p-AMPK), and PGC-1α after red light exposure and after 12 h recovery (N), with densitometric quantification in O (n=6 per group). All experiments were conducted in phenol red-free medium at 37 °C with 5% CO__2__. All assays were independently repeated three times. “n” indicates the number of independent biological samples per group for Western blot quantification. All data are mean ± SEM. Statistical significance was determined by one-way ANOVA with Dunnett’s multiple comparisons test (panels A), or by two-tailed unpaired Student’s *t*-test (panels B, D, G, I-K, M, O). NS, not significant; ^*^P < 0.05; ^**^P < 0.01; ^***^ *P* < 0.001 *vs* Control.

To identify upstream regulators of AMPK activation, we focused on reactive oxygen species (ROS), well-known inducers of AMPK phosphorylation through oxidative stress-sensing mechanisms [37]. Early-life red light exposure induced a 2.0-fold increase in ROS levels, as measured by the ROS-sensitive probe H_2_DCFDA (Fig. 6H), indicating a transient redox shift. In vivo analysis using the transgenic sod-3p::GFP reporter strain revealed a 1.9-fold increase in GFP fluorescence intensity (Supplementary Fig. 11A-C), accompanied by a 2.3-fold increase in superoxide dismutase (SOD) activity. In contrast, catalase (CAT) levels remained unchanged (Supplementary Fig. 11D), suggesting selective activation of the SOD-mediated antioxidant defense system rather than a broad upregulation of all antioxidant enzymes. Notably, malondialdehyde (MDA) levels were unchanged (Supplementary Fig. 11E), excluding the possibility of persistent oxidative damage despite the transient ROS elevation.

In addition to ROS, we investigated whether autophagy contributes to AMPK activation under red-light conditions. Using the *aak-2p::aak-2::GFP* reporter strain, we observed that pharmacological inhibition of autophagy with 3-methyladenine (3-MA, 10 mM) during early-life red-light exposure markedly reduced the AAK-2::GFP fluorescence signal compared with the red-light-only group (Supplementary Fig. 15A, B). This result indicates that autophagy activation is required for the full induction of AMPK in response to early-life red light exposure, suggesting a potential positive feedback loop between autophagy and AMPK signaling in the regulation of mitochondrial quality control.

To establish the functional role of subtoxic ROS, co-treatment with N-acetylcysteine (NAC, 10 mM) or vitamin C (VitC, 5 mM) abolished red light-induced lifespan extension, with median lifespans of 19.2 days for controls and 18.9 days for red light with NAC-treated worms (Fig. 6J, K), implicating ROS as key mediators. In addition, NAC supplementation significantly suppressed the red light-induced increase in the p-AMPK/AMPK ratio (Fig. 6L-O), uncoupling ROS signaling from AMPK activation. Taken together, our results suggest that early-life red light triggers a transient ROS burst and activates autophagy, both converging to promote AMPK activation.

### Conservation of red light’s effects across species—red light-induced mitochondrial proteostasis and AMPK activation delays cellular senescence in human fibroblasts

To assess whether red light-mediated mitochondrial homeostasis is conserved in mammalian cells, we exposed human dermal fibroblasts (HFF-1) to 630 nm red light at an irradiance of 2.5 mW/cm² for varying durations. This regimen was designed to deliver the same total energy density (54 J/cm²) as the nematode protocol when applied for 6 h, thereby enabling cross-species comparison. Cell viability was first assessed using a time-course Cell Counting Kit-8 (CCK-8) assay at 0, 2, 4, 6, 8, 10, and 12 h of irradiation. Red light exposure up to 6 h had no significant effect on cell viability, whereas a slight reduction was observed at ≥8 h (Fig. 7A). Given the critical role of the UPR^MT^ in cytoprotection and longevity, we next quantified mRNA levels of *hsp60*, the mammalian homolog of *hsp-6* in *C. elegans*, a key UPR^MT^ marker. Red light induced a time-dependent activation of *hsp60*, with the most pronounced increase (4.6-fold) occurring at 4 h (Fig. 7B).

To exclude potential confounders affecting light delivery, we tested the influence of phenol red and culture dish lids. Neither factor altered the viability response to red light (Supplementary Fig. 16A, B), although phenol red-free medium slightly improved viability. Thus, subsequent experiments were conducted under phenol red-free conditions without dish lids.

We next evaluated the evolutionary conservation of red light-mediated mitochondrial remodeling and its cytoprotective effects in human cells. HFF-1 fibroblasts were pretreated with red light for 4 h, followed by a 12-hour recovery in the dark, and then challenged with 150 μM H_2_O_2_ for 4 h (Fig. 7C). As shown in Fig. 7D, E, red-light pretreatment significantly reduced the proportion of senescence-associated β-galactosidase (SA-β-gal)-positive cells and increased cell viability compared with dark-treated controls, supporting the hypothesis that early red-light exposure delays the onset of cellular senescence in human fibroblasts.

Given our findings in nematodes, we next examined whether a conserved ROS-AMPK signaling axis mediates these effects in human cells. Live-cell imaging with H_2_DCFDA revealed a 9.5-fold increase in intracellular ROS after 4 h of red-light exposure (Supplementary Fig. 17A, B), indicating rapid activation of redox signaling. Importantly, co-treatment with the ROS scavenger N-acetylcysteine (NAC, 5 mM) completely abolished this red light-induced ROS increase and concomitantly prevented the elevation of phosphorylated AMPK (p-AMPK) levels (Supplementary Fig. 17C, D), demonstrating that AMPK activation in HFF-1 cells is strictly dependent on ROS production.

We next assessed how red light influences mitochondrial properties. Mitochondrial membrane potential (ΔΨm), measured using JC-10, increased by 1.7-fold relative to dark controls (Fig. 7F), reflecting an enhanced bioenergetic state. Interestingly, mtDNA content (mtDNA/nDNA ratio) decreased slightly (Fig. 7G), and expression of *PGC-1α*, a master regulator of mitochondrial biogenesis, remained unchanged both immediately after exposure and following 12 h of recovery (Fig. 7N), suggesting that these effects occur through a biogenesis-independent pathway.

Consistently, the ratio of MTCO1 (mtDNA-encoded) to ATP5A (nDNA-encoded), two representative subunits of the oxidative phosphorylation system. This ratio was significantly increased, indicating enhanced mitochondrial protein expression relative to nuclear-encoded components (Fig. 7H-K). In parallel, the expression of CLPP (caseinolytic mitochondrial matrix peptidase proteolytic subunit), a key mitochondrial protease involved in protein quality control, was upregulated by 1.3-fold (Fig. 7L, M), further supporting MQC activation.

Interestingly, although total AMPK activity did not change immediately post-irradiation, phosphorylation at Thr172 (p-AMPK) increased significantly 12 h after red light exposure (Fig. 7N, O). Taken together with the NAC results, these data suggest that red light triggers a ROS-dependent AMPK activation during exposure, which persists beyond the light period and likely contributes to sustained mitochondrial remodeling and delayed senescence.

## DISCUSSION

Non-pharmacological physical interventions such as exercise and dietary restriction are widely recognized for their anti-aging benefits. Growing evidence suggests that modulating light exposure and circadian rhythms can improve metabolic health and promote longevity, positioning light as a promising non-invasive strategy for healthy aging. In this study, using the non-visual model organism *C. elegans*, we demonstrate that early-life exposure to red light (630 nm) markedly extends lifespan and mitigates multiple age-associated phenotypes. This pro-longevity effect is dose-dependent and confined to a discrete early-life developmental window. Furthermore, we show that red light-induced lifespan extension is mediated via a ROS-AMPK signaling axis that enhances mitochondrial quality control (MQC) and homeostasis. This mitochondria-centric protective mechanism was further validated in human dermal fibroblasts, underscoring its evolutionary conservation and translational relevance. Together, our findings reveal a mitochondria-centric adaptive response initiated by early-life red light exposure in non-visual systems, and provide a conceptual framework for developing non-invasive, evolutionarily conserved interventions for delaying aging. In this study, we systematically evaluated the effects of various visible light wavelengths on the lifespan and healthspan of *C. elegans* under indoor lighting conditions (200 lux). Short-term exposure to red, blue, green, or white light extended lifespan to varying extents; however, only low-intensity red light conferred sustained longevity benefits without adversely affecting growth or neuromuscular function. In contrast, although blue light elicited a modest lifespan extension, it impaired healthspan, consistent with previous reports that continuous visible light exposure disrupts *C. elegans* development [38].

These wavelength-specific effects likely arise from distinct underlying photobiological mechanisms. In mammals, blue light is primarily sensed via retinal photoreceptors and intrinsically photosensitive retinal ganglion cells (ipRGCs), while red light is mainly absorbed by mitochondrial cytochrome c oxidase (COX), modulating energy metabolism [39, 40]. In the non-visual organism *C. elegans*, short-wavelength light is detected by the LITE-1 receptor [41]; however, no red light-specific photoreceptor has been identified to date. Notably, red light exposure significantly enhanced lifespan and activated mitochondrial quality control (MQC), suggesting that red light may be perceived through metabolism-linked or alternative phototransduction mechanisms. These findings raise the possibility that non-visual systems harbor compensatory or parallel pathways to distinguish between different light wavelengths.

We identified a precise dose and temporal window in which red light exposure extends the lifespan of *C. elegans*. Low-intensity and short-duration illumination during early development significantly prolonged lifespan. However, when exposure exceeded five days or was delayed until adulthood, the beneficial effect was lost and, in some cases, even reversed, despite higher cumulative photon input. This biphasic response reflects a hallmark of PBM, where moderate stimulation confers benefits whereas excessive exposure becomes detrimental [42]. These findings also highlight a critical developmental period characterized by enhanced mitochondrial and epigenetic plasticity. This concept is further supported by prior studies demonstrating that early-life nutritional, thermal, and oxidative stress can durably reprogram metabolic trajectories through organelle remodeling and proteostasis regulation [43-47].

Our results are consistent with the hormesis paradigm, particularly the mitohormesis model, which proposes that moderate levels of reactive oxygen species (ROS) can activate adaptive stress responses that ultimately promote longevity [44, 48]. Notably, red light exposure in adulthood failed to yield similar benefits, reinforcing the view that early developmental stages represent a critical window for reprogramming aging trajectories. Supporting this notion, previous studies have shown that early activation of key metabolic regulators such as AMPK and mTOR can exert long-term effects on lifespan, in line with our observations [46, 47].

Mechanistically, we found that red light induces mild generation of reactive oxygen species (ROS), which activates AMPK phosphorylation and orchestrates a series of downstream mitochondrial quality control (MQC) pathways. These include the UPR^MT^, mitochondrial dynamics, mitophagy, and proteostasis remodeling. Together, these adaptive processes promote mitochondrial functional remodeling, restore homeostasis without causing sustained damage, and ultimately delay aging—hallmarks of mitohormesis. Similar responses have been observed under caloric restriction, thermal stress, and exercise conditions [49-51]. Importantly, the mechanism of our red light intervention is distinct from conventional behavioral or pharmacological anti-aging strategies, which often target systemic metabolic or nutrient-sensing pathways [52]. While previous PBM studies have primarily emphasized enhanced ATP production, calcium flux regulation, or activation of signaling cascades such as AKT/GSK3β and mTOR [53-55], our findings reveal a distinct redox-dependent mechanism. Specifically, the ROS-AMPK axis emerges as a central regulatory node for red light-induced mitochondrial reprogramming.

MQC plays a central role in maintaining proteostasis and mitigating the pathology of neurodegenerative diseases [56]. In a polyglutamine (polyQ) aggregation model, we found that early-life exposure to red light significantly reduced polyQ puncta accumulation in adult *C. elegans*. PolyQ aggregation is widely recognized as a molecular hallmark of Huntington’s disease and several other neurodegenerative disorders [57]. Previous studies have shown that such protein aggregates can be alleviated through chemical chaperones, autophagy activators, or thermal stress [58]. To our knowledge, this is the first study to demonstrate that physiological-level red light exposure alone is sufficient to produce a comparable effect. These findings suggest that MQC-based photonic interventions may hold therapeutic potential for protein aggregation-related diseases.

To further validate the molecular basis of red light-induced longevity, we conducted a cross-species comparison in HFF-1 human dermal fibroblasts. Interestingly, while red light activated AMPK in both models, the activation kinetics differed markedly: in *C. elegans*, AMPK phosphorylation occurred rapidly after exposure, whereas in HFF-1 cells, it was delayed by approximately 12 hours. This disparity may reflect species-specific traits or differences in the spatial organization of intracellular signaling. As an intact organism, *C. elegans* can coordinate stress perception and signal transduction rapidly across tissues, whereas in mammalian cells, AMPK activation may depend on slower transcriptional, translational, or post-translational processes [59]. In addition, the timing of AMPK activation depends on the type of stress and its subcellular localization; for example, glucose deprivation can cause a delay of several hours, whereas inhibition of oxidative phosphorylation can induce rapid activation within minutes [60]. These observations suggest that red light-induced metabolic stress may represent a mild, gradual ROS signaling process, with AMPK temporal dynamics shaped by a combination of species, cell type, and subcellular organization. Overall, AMPK activation by red light appears to be evolutionarily conserved, while the delayed response in human fibroblasts indicates that downstream MQC and metabolic remodeling may persist beyond the exposure period. This feature underscores the importance of considering the temporal scale of cellular responses when designing photobiomodulation strategies for human application.

## Limitations

Although this study uncovered the mechanism by which red light delays aging via the ROS-AMPK-MQC axis in model organisms and human-derived cells, demonstrating strong cross-species conservation, several aspects still warrant further exploration. First, the effectiveness and time window of this mechanism require further validation in mammalian in vivo models to determine its therapeutic potential in complex physiological systems. Second, while red light significantly delays neuromuscular degeneration and reduces the expression of polyglutamine proteins, its effects on other behavioral and cognitive functions, such as habituation, thermotaxis, and learning abilities, have not yet been evaluated. This limitation hinders a comprehensive understanding of the role of red-light intervention in promoting functional healthspan, particularly in the context of its potential for preventing neurodegenerative diseases. Finally, although this study clarifies the role of AMPK activation in red light intervention, the upstream photoreceptor genes and their interplay with tissue metabolic status require further exploration. In the future, combining animal behavior assessments, tissue function evaluations, and histological techniques is expected to further refine the mechanistic mapping of this light-regulated anti-aging strategy.

## Conclusion

In summary, the present study systematically reveals the mechanism of red-light delaying aging through the activation of the ROS-AMPK-MQC signaling axis during early life, addressing a critical knowledge gap in understanding how photobiomodulation regulates aging in vivo and the significance of temporal windows. By comprehensively elucidating the time dependency, dosage effects, and mitochondrial remodeling induced by red light interventions, we have established for the first time a causal link between red light interventions and MQC, further validating its evolutionary conservation in mammalian cells. These findings provide mechanistic insights supporting the development of non-invasive early-life interventions, facilitating the long-term preservation of mitochondrial health and deceleration of the aging process.

## Supplementary Materials

The Supplementary data can be found online at: www.aginganddisease.org/EN/10.14336/AD.2025.0791.

## Data Availability

All data that support the findings in this study is available from the authors on reasonable request.
