# Supplementary material for: Early-Life Red Light Exposure Extends Lifespan in C. elegans through ROS-AMPK-Driven Mitochondrial Reprogramming
Source: Aging Dis. 2026 Sep 9;17(6):3337–59. doi: 10.14336/AD.2025.0791 (PMC13653719; doi:10.14336/AD.2025.0791)
Supplement: Supplementary file 1 [file AD-17-6-3337-s.pdf]

SUPPLEMENTARY DATA

Early-Life Red Light Exposure Extends Lifespan in *C. elegans* through ROS–AMPK-Driven Mitochondrial Reprogramming

Yao Zhu, Xin Lin, Huiting Wang, Mingming Song, Tao Yang, Yi Jiao, Chang Liu

© 2025. Zhu Y et al. Published online at http://www.aginganddisease.org/EN/10.14336/AD.2025.0791

SUPPLEMENTARY DATA

Materials and methods

Light experiments and dose selection

Worms cultured on NGM plates were exposed to specific wavelengths of light in a custom-designed illumination incubator, with lids removed. Narrow-band LEDs were used to emit the following wavelengths: red (625-635 nm), blue (460-475 nm), green (520-533 nm), and white (380-750 nm). For the dark control group, LED panels were covered, and lights were turned off during the experiment. The LED output intensity and wavelength were calibrated using a validated spectroradiometer (Hopoocolor Technology, China) and recorded as both illuminance (lux) and spectral irradiance (mW/cm^2). All multi-wavelength comparison groups were maintained at 200 lux with identical plate–LED geometry.

For the early-life red-light regimen in lifespan and mechanistic assays, worms were exposed to 200 lux (0.25 mW/cm^2) continuously for 60 h, corresponding to an energy density of 54 J/cm^2 (calculated as $H = \text{irradiance} \times \text{time}$). Plates were positioned at equal distances from the LED panels to ensure uniform exposure, and no lids or filters were used to avoid light attenuation.

For mammalian cell irradiation, we selected a dose that matched the nematode regimen in total energy delivery and was supported by previous photobiomodulation studies [1]. The LED panels of our previous cell illumination device were replaced with the same light source used for nematode experiments. HFF-1 cells were exposed to 2.5 mW/cm^2 (with lids removed) for 6 h, yielding an identical total energy density of 54 J/cm^2. This parameter refers to the light intensity used by Zhao et al. in mammalian cells [1], which is within the established photobiological regulation range. In our experimental protocol, preliminary tests have confirmed that this dose has no adverse effect on the survival rate of HFF-1 cells.

Temperature Measurements

Under four light conditions, a Bluetooth-enabled thermo-hygrometer (Jiali Technology, China) was placed on NGM agar plates to record the surface temperature every hour until the light exposure period ended. Four plates were used for each condition, and three independent measurements were performed.

Fluorescence Imaging Analysis

Worms were randomly selected, placed on 2% agar pads, anesthetized with 5 mg/mL levamisole, and imaged under DIC or GFP fluorescence. GFP fluorescence intensity was quantified using ImageJ software. Images were captured with fixed exposure times to avoid saturation, ensuring accurate fluorescence comparisons. To minimize levamisole-induced artifacts, slides were freshly prepared and imaging completed within 15 minutes.

For strain SJ4103 (zcIs14 [*myo-3::GFP* (mit)]), body wall muscle regions were visualized using a 63× oil immersion objective on a confocal microscope (LSM800, Zeiss, Germany). Approximately 20-30 worms were imaged per condition. Images were processed and analyzed with Fiji (ImageJ).

All fluorescence signals in this study were derived from genetically encoded GFP or mCherry reporters integrated into the endogenous loci of target genes in transgenic *C. elegans* strains, rather than from antibody-based immunostaining.

© 2025. Zhu Y et al. Published online at http://www.aginganddisease.org/EN/10.14336/AD.2025.0791

SUPPLEMENTARY DATA

Supplementary Table 1. List of strains used in this study.

Strain	Genotype	Source
N2	*C. elegans* wild type isolated from Bristol, UK	Caenorhabditis Genetics Center
OG412	drIs20 [vha-6p::Q44::YFP + rol-6(su1006) + pBluescript II]	OG412 was a general gift from Professor Caishiqing (Institute of Neuroscience and State Key Laboratory of Neuroscience, Chinese Academy of Sciences).
SJ4103	zcIs14 [myo-3::GFP(mit)]	SJ4103 was a general gift from Professor dongdeli (Department of Pharmacology, College of Pharmacy, China Pharmaceutical University).
SJ4100	zcIs13 [hsp-6p::GFP + lin-15(+)]	Caenorhabditis Genetics Center
SJ4005	zcIs4 [hsp-4::GFP] V	
TJ375	gpIs1 [hsp-16.2p::GFP]	
DA2123	adIs2122 [lgg-1p::GFP::lgg-1 + rol-6(su1006)]	
MAH145	sqEx4 [atg-18p::atg-18::GFP + rol-6(su1006)]	
MAH349	sqIs35 [sqst-1p::sqst-1::GFP + unc-122p::RFP]	
CL2166	dvIs19 [(pAF15)gst-4p::GFP::NLS] III	
CF1553	muIs84 [(pAD76) sod-3p::GFP + rol-6(su1006)]	
TJ356	zIs356 [daf-16p::daf-16a/b::GFP + rol-6(su1006)]	
LD1	ldIs7 [skn-1b/c::GFP + rol-6(su1006)]	
WBM60	uthIs248 [aak-2p::aak-2(genomic aa1-321)::GFP::unc-54 3'UTR + myo-2p::tdTOMATO]	
RB754	aak-2(ok524) X	
MAH235	sqIs19 [hlh-30p::hlh-30::GFP + rol-6(su1006)]	
MAH215	sqIs11 [lgg-1p::mCherry::GFP::lgg-1 + rol-6]	

© 2025. Zhu Y et al. Published online at http://www.aginganddisease.org/EN/10.14336/AD.2025.0791

SUPPLEMENTARY DATA

Supplementary Table 2. Statistics for lifespan experiments performed in this study.

Supplementary Table 2: Statistics for lifespan experiments performed in this study						
Strain-Condition	Mean lifespan ± SEM (days)	Median (days)	Total animals	p-Value		
Fig 1						
panel B				*vs* Control	*vs* WL	
Control	19.78±0.18	20	328			
WL	20.46±0.22	21	301	0.0014		
RL	20.15±0.24	23	279	<0.0001	<0.0001	
BL	21.66±0.28	20	299	0.0236		
GL	20.19 ± 0.21	20	305	0.0496		
panel H				*vs* Control		
0 h	19.27 ± 0.26	19	177			
1 h	18.78 ± 0.27	18	120	0.1075		
2 h	23.37 ± 0.42	23	134	<0.0001		
4 h	20.93 ± 0.36	20	108	0.0005		
6 h	22.64 ± 0.46	23	116	<0.0001		
8 h	23.69 ± 0.43	23	180	<0.0001		
panel I				*vs* Control		
0 d	17.93 ± 0.13	18	304			
1 d	20.03 ± 0.28	21	190	<0.0001		
2 d	19.31 ± 0.32	22	197	<0.0001		
3 d	19.65 ± 0.38	21	101	<0.0001		
4 d	18.71 ± 0.30	18	117	0.0028		
5 d	18.69 ± 0.16	18	250	<0.0001		
6 d	16.86 ± 0.18	17	106	<0.0001		
panel J				*vs* Control		
0 h	18.50 ± 0.27	18	167			
24 h	18.58 ± 0.22	18	245	0.999		
48 h	17.82 ± 0.20	18	150	0.5638		
60 h	17.90 ± 0.20	18	200	0.1368		
72 h	18.07 ± 0.21	18	248	0.0322		
96 h	18.39 ± 0.21	18	303	0.2206		
Fig 4						
panel H				*vs* L4440-control		
L4440-control	21.0 ± 0.37	23	201			
L4440-RL	22.6 ± 0.36	23	210	0.001		
panel I				*vs atfs-1*-control		
atfs-1-control	20.42 ± 0.28	22	168			
atfs-1-RL	20.91 ± 0.26	22	182	0.1534		
panel J				*vs ubl-5*-control		
ubl-5-control	14.90 ± 0.27	15	132			
ubl-5-RL	14.74 ± 0.24	15	184	0.6497		
panel K				*vs lin-65*-control		
lin-65-control	17.20 ± 0.21	17	297			
lin-65-RL	18.16 ± 0.25	19	190	0.0241		
Fig 5						
panel K				*vs* Control (-)	*vs* Control (+3-MA)/(RL-)	
Control (-)	18.35 ± 0.17	18	153			
RL (-)	19.51 ± 0.17	19	210	<0.0001		
Control (+3-MA)	17.98 ± 0.24	17	102			
RL (+3-MA)	18.17 ± 0.16	18	186		NS/<0.0001	
Fig 6						
panel G					*vs aak-2* DD	*vs* N2 RL
N2 DD	18.05 ± 0.22	18	124			
N2 RL	19.5 ± 0.24	21	159	<0.0001		

© 2025. Zhu Y et al. Published online at http://www.aginganddisease.org/EN/10.14336/AD.2025.0791

aak-2 DD	17.48 ± 0.34	19	149			
aak-2 RL	17.93 ± 0.32	19	167		0.1564	0.1777
panel J				*vs 5 mM NAC + DD*	*vs 10 mM NAC + DD*	
5 mM NAC + DD	18.77 ± 0.27	20	132			
10 mM NAC + DD	19.32 ± 0.38	21	109			
5 mM NAC + RL	20.63 ± 0.27	21	125	<0.0001		
10 mM NAC + RL	18.51 ± 0.41	20	97		0.2177	
panel K				*vs 5 mM VC + DD*	*vs* 10 *mM VC + DD*	
5 mM VC + DD	19.37 ± 0.24	20	132			
10 mM VC + DD	19.46 ± 0.20	20	172			
5 mM VC + RL	19.92 ± 0.16	20	172	0.13		
10 mM VC + RL	17.69 ± 0.25	18	133		<0.0001	
FigS4						
panel A				*vs* Control		
Control	20.47 ± 0.27	22	125			
20.6°C	20.33 ± 0.25	21	160	0.841		
panel B				*vs* Control		
Control	19.78 ± 0.36	21	111			
RL	20.86 ± 0.37	22	115	0.0326		

WL: white light; RL: Red light; BL: Blue light; GL: Green light; DD: Continuous darkness; 3-MA: 3-Methyladenine; VC: vitamin C; NAC: N-Acetylcysteine.

© 2025. Zhu Y et al. Published online at http://www.aginganddisease.org/EN/10.14336/AD.2025.0791

SUPPLEMENTARY DATA

Supplementary Table 3. Primer sequence of target genes used in this study.

Gene Name	Species	Forward 5'-3'	Reverse 5'-3'
act-1	*C. elegans*	tgctgatcgtatgcagaagg	tagatcctccgatccagacg
fzo-1	*C. elegans*	agtcggcattcccctgattccg	tctgcaggttgaaggttcagaaggc
opa-1	*C. elegans*	cttggccatccattctgccca	tcgcggctagaacgtggtatga
cts-1	*C. elegans*	ctcgacaacttcccagataacc	ggtacaggttgcgatagatgatagc
cox-4	*C. elegans*	gccccaattcgcgccaagga	aggttggcggcagttctggg
hxk-1	*C. elegans*	gtgcgacgagtactttctcaactg	ctagagatgacgtcacacacttctc
pyc-1	*C. elegans*	tccaactactcctcttgctactgac	gtgatcatacatcctggtctactgc
hsp-60	*C. elegans*	agggattcgagagcattcgtcaag	tgtggcgacttgagcgatctcttc
hsp-4	*C. elegans*	cagatgaaaactcaaatcgcc	ggttgcttccgagccactcaa
xbp-1s	*C. elegans*	cgtgcctttgaatcagcagtg	cgaggtgtccatcttcttgtt
hsp-70a	*C. elegans*	cggtatttatcaaaatggaaaggtt	tacgagcggcttgatctttt
hsp-16.2	*C. elegans*	tgcagaatctctccatctgagt	tggtttaaactgtgagacgttga
hsp12.3	*C. elegans*	ggccattccagaaaggaga	caatgttcttcacatcaatctcg
hsp-16.11	*C. elegans*	ggctcagatggaacgtcaa	tggcaaacttttgatcattgtta
hsp-16.41	*C. elegans*	tcttggacgaactcactgga	agagacatcgagttgaaccga
hsp-16.49	*C. elegans*	ctcatgctccgttctccatt	gagttgtgatcagcatttctcca
drp-1	*C. elegans*	cattcaggatggatcggttgacgc	caggattggccaagttcttctc
lin-65	*C. elegans*	agaagagctccaccattggc	tggtagacgtgtcgaatcgc
ubl-5	*C. elegans*	ttgaaatcacagtaaacgatcgac	agctcgaaattgaatccctcgt
atfs-1	*C. elegans*	ggtttgcggcagagatccta	agctgatcgatcgccgattt
dve-1	*C. elegans*	ctcgaatccctctttggcga	cgtttccagcgtatcaagcc
clpp-1	*C. elegans*	aacagtccaggcggcagtgt	ttcaccagccgagtggtggt
atg-18	*C. elegans*	aaatggacatcggctctttg	tgatagcatcgaaccatcca
bec-1	*C. elegans*	tcgagctcccactctttggcg	tgacaccattgtcaaccagtg
lgg-1	*C. elegans*	acccagaccgtattccagtg	acgaagttggatgcgttttc
sqst-1	*C. elegans*	tggctgctgcatcatccgct	tcaatcgtgccgagaccggg
vps-11	*C. elegans*	tccgcttgtcgtcctggagc	tcacacgccgagcacttggt
lmp-1	*C. elegans*	atccgccaccgcttcgcatt	tcgagctcccactctttggcg
vha-15	*C. elegans*	cgaggttcgttccggacgtctt	cctcggcagtcaggagacgc
vha-16	*C. elegans*	aggcgctgactcgcggactt	tggtctctggtgaagagttccggtg
pink-1	*C. elegans*	aagcaccagaaattgcgacg	acgagatgggagtgctggta
pdr-1	*C. elegans*	gatcttccatcgtcatcataggg	cagggtgcaatagagtggtacaaga
dct-1	*C. elegans*	gcaaaagccgtctcaaaccc	acccacgattctgacatacca
nucDNA	*C. elegans*	gccgactggaagaacttgtc	gcggagatcaccttccagta
mtDNA	*C. elegans*	agcgtcatttattgggaagaagac	aagcttgtgctaatcccataaatgt
cls-1	*C. elegans*	ttcgtccccatcaacttcga	tgctaggcttgaacggagaa
18s	Human	ctaccacatccaaggaaggca	tttttcgtcactacctccccg
hsp-60	Human	agcccgagtgagatgaggag	atgcttcggttacccacagtc
slco2B1	Human	ggatgtggagaggacttgct	ctgccacaagaacaaacgga
nd1	Human	tcctgccatcatgacccttg	ctgcggcgtattcgatgttg

© 2025. Zhu Y et al. Published online at http://www.aginganddisease.org/EN/10.14336/AD.2025.0791

SUPPLEMENTARY DATA

SUPPLEMENTARY DATA

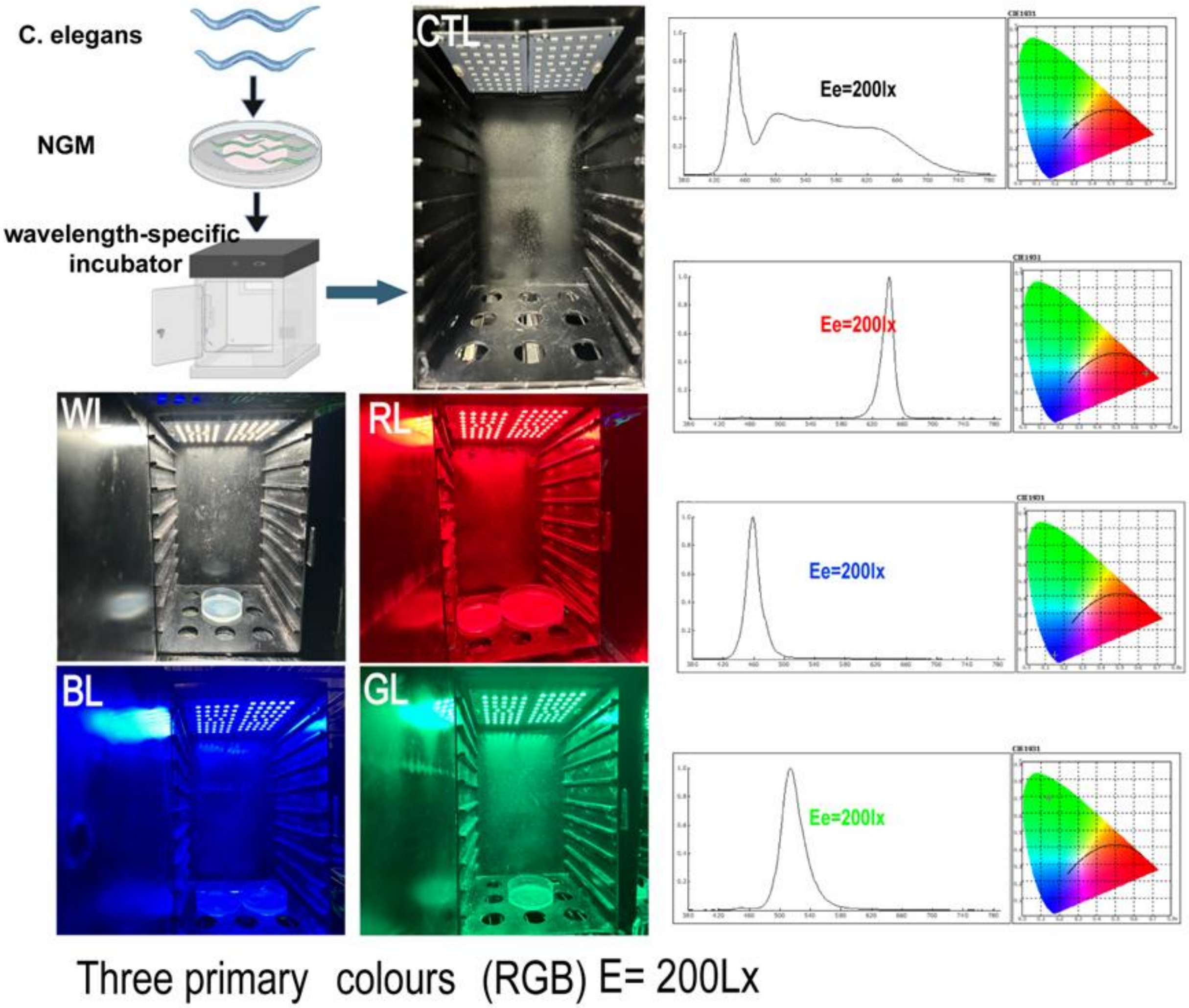

Supplementary Figure 1. Custom built wavelength specific illumination system for *C. elegans* lifespan assays. Left: Schematic representation and photographs of the custom-designed light-controlled incubator (15 × 15 × 20 cm³) equipped with modular LED panels. The system supports interchangeable monochromatic light modules, including red (630 ± 5 nm), blue (465 ± 5 nm), green (525 ± 5 nm), and white (380–750 nm) light, providing uniform overhead illumination. Photon flux density was maintained at 200 lux, with a fixed 20 cm distance between the light source and the samples. All culture dishes remained uncovered during exposure to ensure consistent light delivery. Synchronized L1-stage *C. elegans* were cultured on NGM plates within this custom-built steel incubator under monochromatic (red, blue, green) or full-spectrum white light. Worms were exposed without covers, maintaining a constant 20 cm distance from the light source. The surface temperature of the agar medium was monitored continuously to control for thermal artifacts (Fig S3 in Supporting Information). Temperature fluctuations were minimal (~0.4 ± 0.2°C compared to dark controls), confirming negligible thermal effects during light exposure. Right: Spectral profiles and chromaticity diagrams of each light source, measured with an Ocean Insight USB4000 spectrometer. Emission peaks were centered at 630 nm (red), 468 nm (blue), 527 nm (green), and broadband for white light. The full-width at half-maximum (FWHM) for each monochromatic light was maintained below 15 nm, ensuring wavelength specificity.

© 2025. Zhu Y et al. Published online at http://www.aginganddisease.org/EN/10.14336/AD.2025.0791

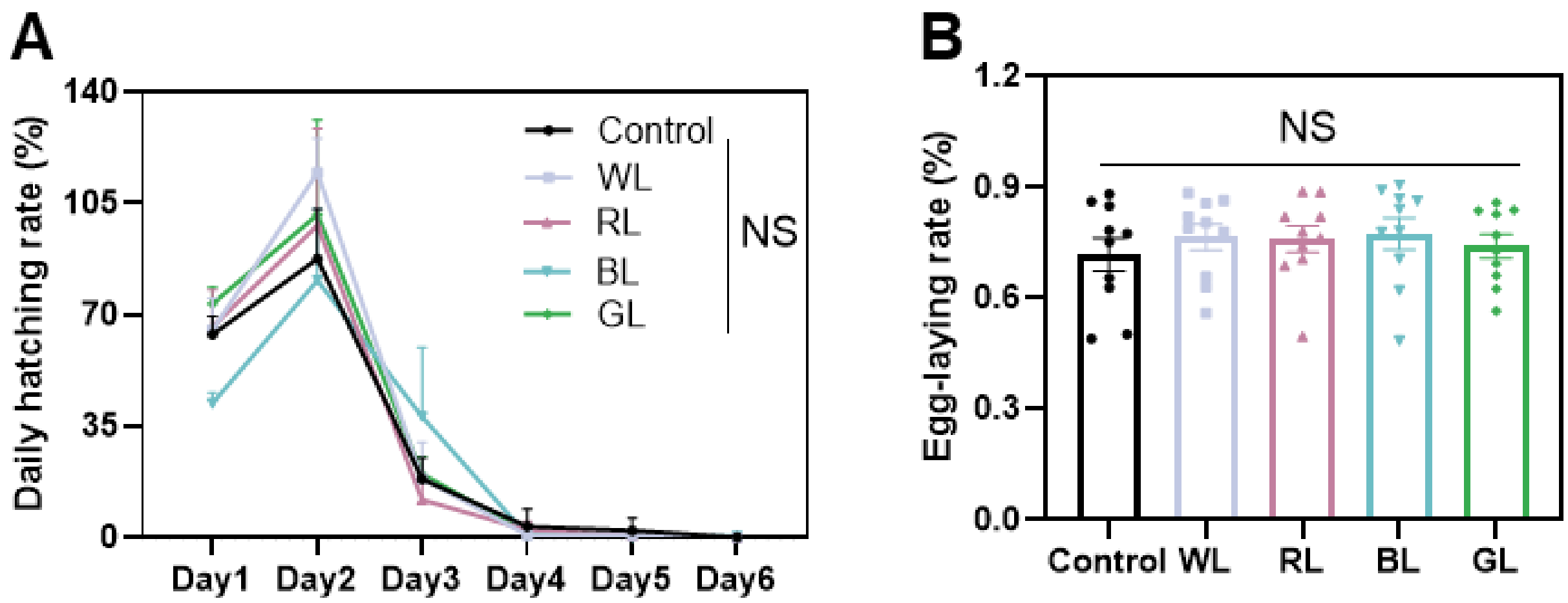

Supplementary Figure 2. Daily and cumulative hatching rates of *C. elegans* under defined spectral conditions. (A), Daily hatching rates (%) of wild-type (WT) N2 worms continuously exposed to monochromatic red, green, or blue light, or full-spectrum white light, from the L1 stage to the young adult stage. No significant delay or acceleration in hatching timing was observed under any light condition compared to the dark control. (B), Cumulative hatching rates of WT worms following exposure to different light wavelengths (n = 9 per group). All assays were independently repeated three times. "n" indicates the number of individual worms per group (biological replicates). Data are mean ± SD. Statistical significance was determined by one-way ANOVA with Dunnett's multiple comparisons test. NS, not significant; * $P < 0.05$ *vs* Control.

© 2025. Zhu Y et al. Published online at http://www.aginganddisease.org/EN/10.14336/AD.2025.0791

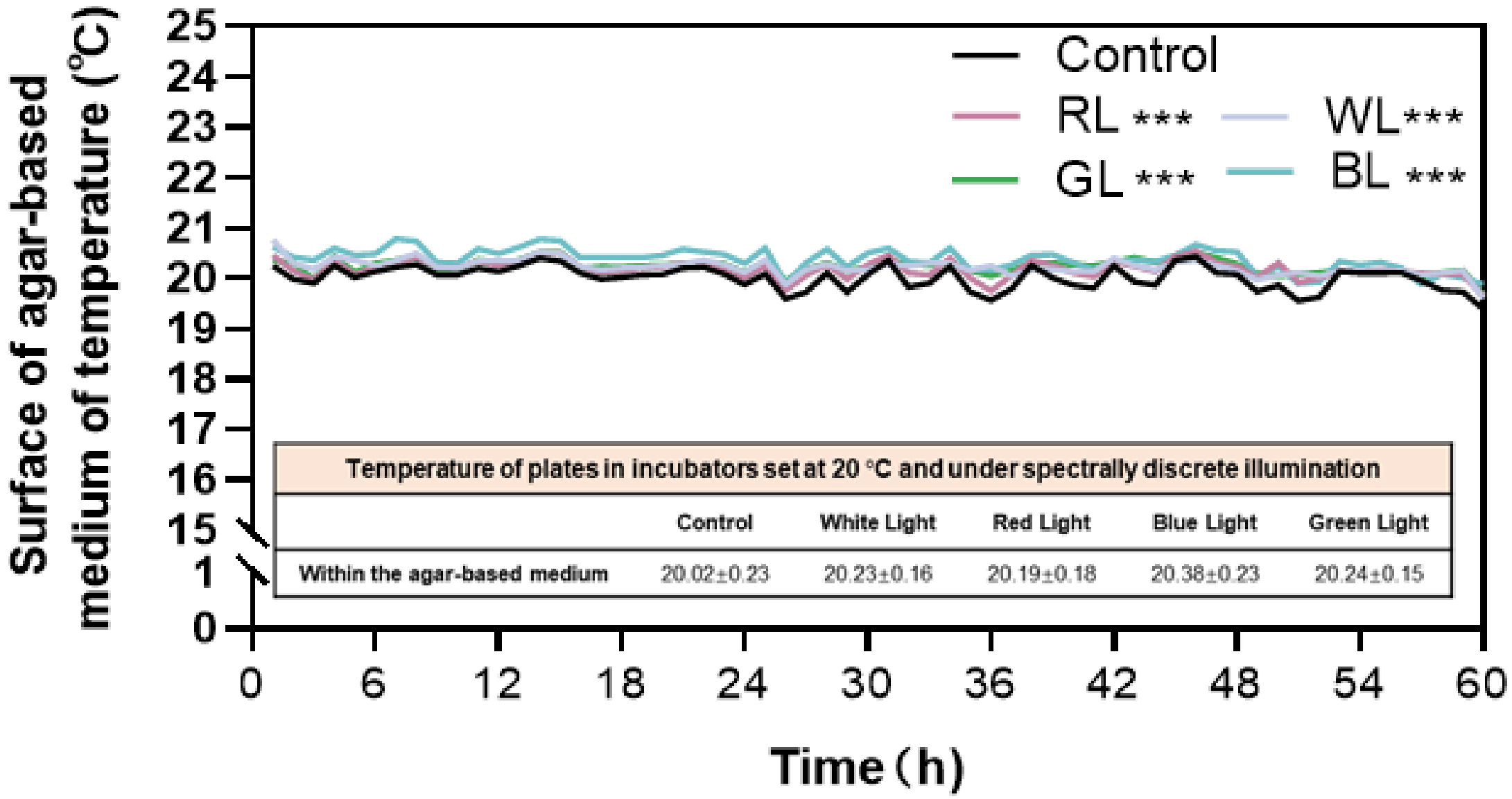

Supplementary Figure 3. Surface temperature stability of agar plates under different light conditions. Surface temperature of NGM plates was continuously monitored over 60 hours under red, blue, green, or white light using a Bluetooth-enabled thermometer. Incubators were maintained at a constant ambient temperature of 20 °C. Hourly temperature recordings showed minimal variation across spectral conditions. Assay was independently repeated three times. Data are mean ± SEM. Statistical significance was determined by one-way ANOVA followed by Dunnett's multiple comparisons test. *** *P*<0.001 *vs* Control.

© 2025. Zhu Y et al. Published online at http://www.aginganddisease.org/EN/10.14336/AD.2025.0791

SUPPLEMENTARY DATA

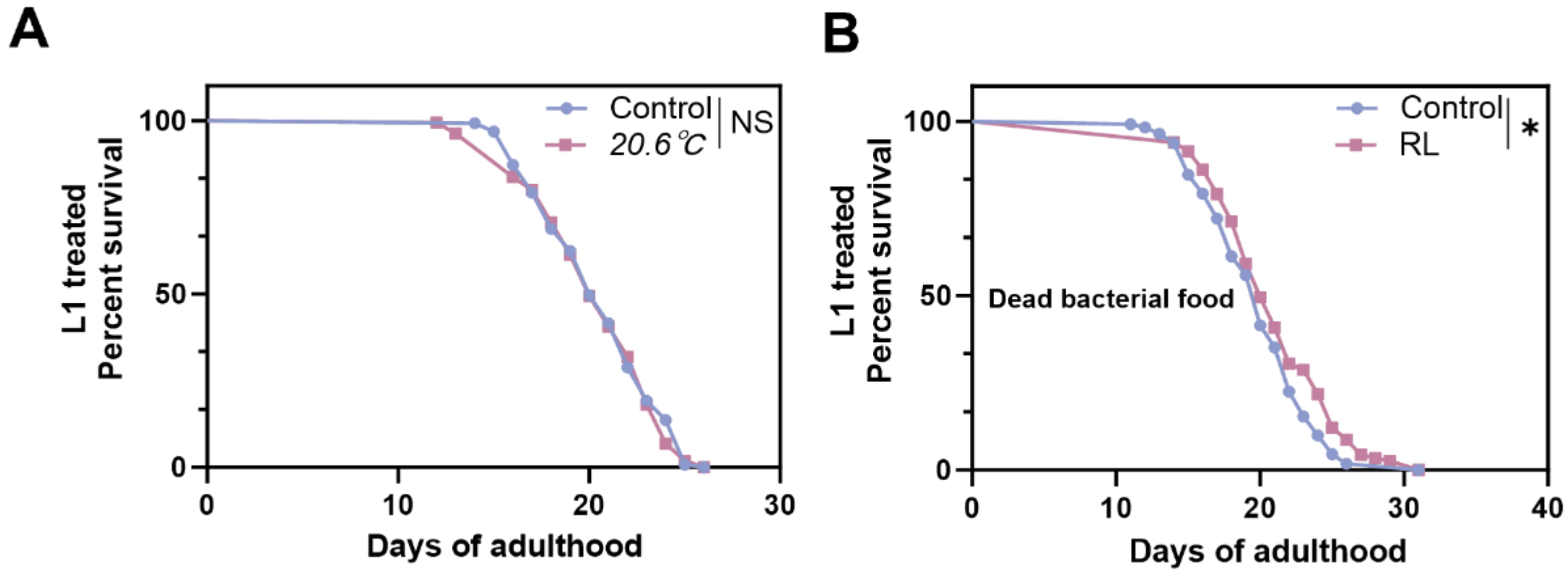

Supplementary Figure 4. Temperature and live bacterial metabolites cannot fully explain the prolongation of nematode lifespan caused by red light exposure. (A), Survival of WT (N2) worms maintained at 20.6 °C from the L1 to young adult stage to simulate potential thermal effects of red -light exposure, then transferred to 20 °C for lifespan assessment from the young adult stage onward (n = control: 125; 20.6°C: 160). (B), Survival of WT worms fed heat-killed *E. coli* OP50 during light exposure from L1 to young adult stage and switched to live *E. coli* OP50 from the young adult stage onward to control for potential metabolic differences caused by light-induced changes in bacterial activity (n = control: 111; RL: 115). All assays were independently repeated three times. “n” indicates the number of individual worms per group (biological replicates). Data are mean ± SEM. Statistical significance was determined by log-rank (Mantel–Cox) test. NS, not significant; * $P < 0.05$ *vs* control.

© 2025. Zhu Y et al. Published online at http://www.aginganddisease.org/EN/10.14336/AD.2025.0791

SUPPLEMENTARY DATA

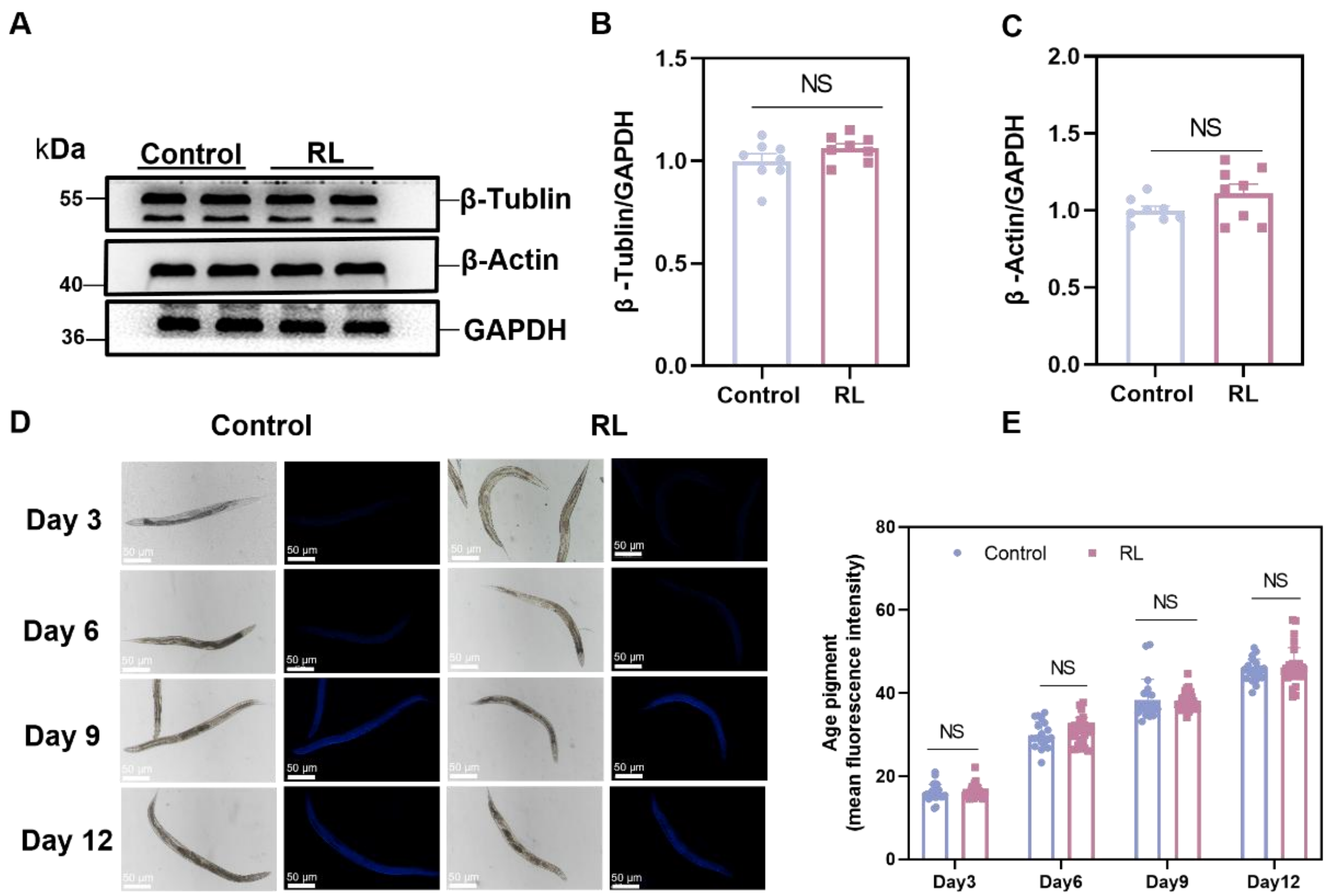

Supplementary Figure 5. Red light exposure during early adulthood does not alter cytoskeletal protein levels or lipofuscin accumulation in aged worms. (A–C), Western blot analysis of cytoskeletal proteins in day 6 adult WT worms following red light exposure from the L1 to young adult stage. Protein levels of β-actin and β-tubulin were assessed Fig A, with densitometric quantification shown in Fig B and C (n = 8). GAPDH was used as the loading control. (D, E), Representative fluorescence images (D) and quantification (E) of lipofuscin autofluorescence in day 3, 6, 9, and 12 WT worms after early-life red light exposure (n = 20 per group; Scale bar, 50 µm). Assays were independently repeated three times (D, E); data in panels A–C represent four independent biological replicates. "n" indicates the exact number of worms per group in panel (E), while n = 4 denotes four independent biological samples for Western blot quantification. Data are mean ± SEM (B, C) or mean ± SD (E). Statistical significance was determined by two-tailed unpaired Student's *t*-test. NS, not significant.

© 2025. Zhu Y et al. Published online at http://www.aginganddisease.org/EN/10.14336/AD.2025.0791

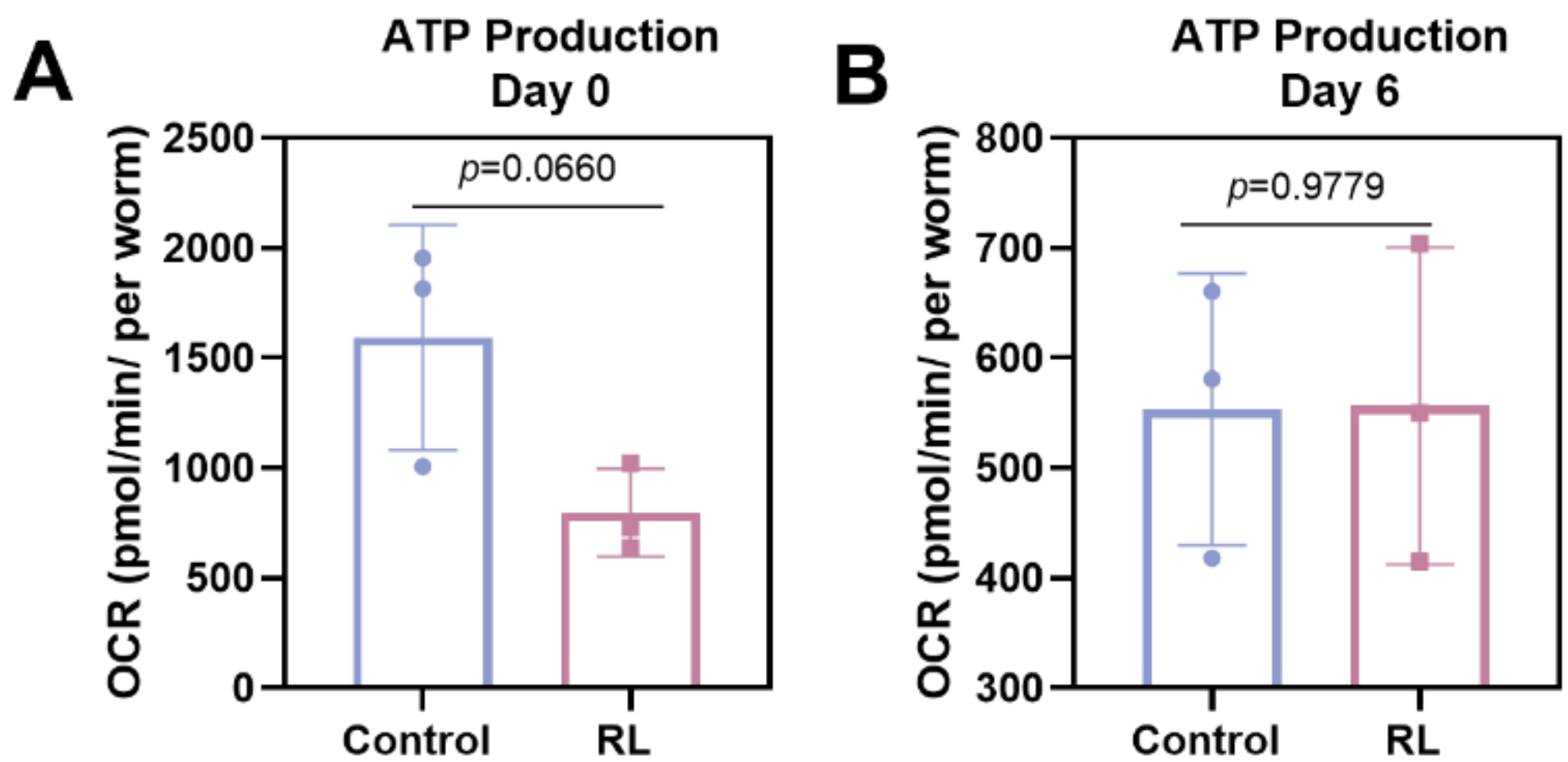

Supplementary Figure 6. Red light exposure did not significantly affect mitochondrial ATP-linked respiration. (A-B), Oxygen consumption rate (OCR) was measured in whole worms immediately after red light withdrawal (day 0) and on day 6 of adulthood. After determining basal OCR, DCCD and FCCP were sequentially injected to calculate ATP-linked respiration (defined as basal OCR minus DCCD-inhibited OCR) (n = control: 17, 19, 20 (days 0); 20, 19, 15 (days 6); RL: 18, 17, 16 (days 0); 21, 21, 16 (days 6)). All assays were independently repeated three times. "n" indicates the number of independent biological samples per group. Data are mean ± SEM. Statistical significance was determined by two-tailed unpaired Student's *t*-test. NS, not significant.

© 2025. Zhu Y et al. Published online at http://www.aginganddisease.org/EN/10.14336/AD.2025.0791

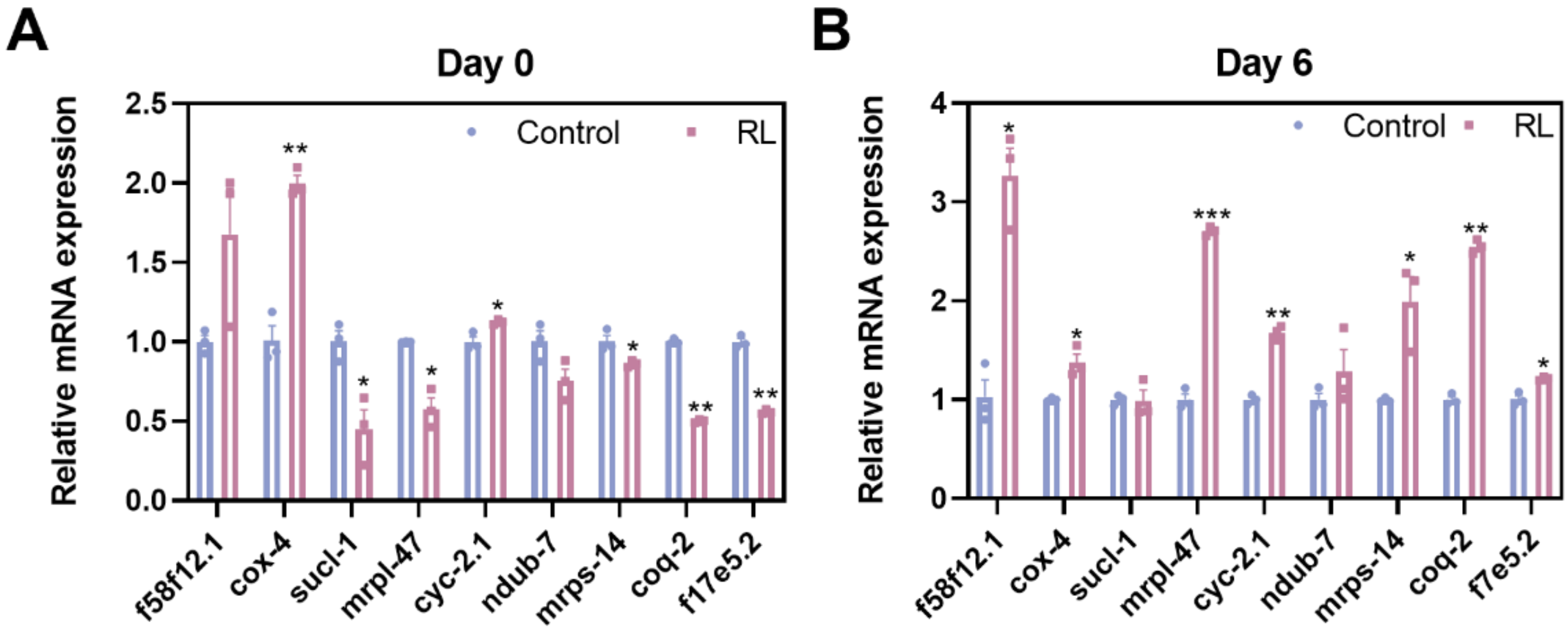

Supplementary Figure 7. Red light exposure transiently alters the expression of nuclear-encoded mitochondrial genes. (A, B), Relative mRNA expression of nuclear-encoded mitochondrial genes compared with control in wild type (WT) worms on day 0 (A) and day 6 (B) post exposure. All assays were independently repeated three times. Data are mean ± SEM. Statistical significance was determined by two-sided Student's *t*-test. NS, not significant.

© 2025. Zhu Y et al. Published online at http://www.aginganddisease.org/EN/10.14336/AD.2025.0791

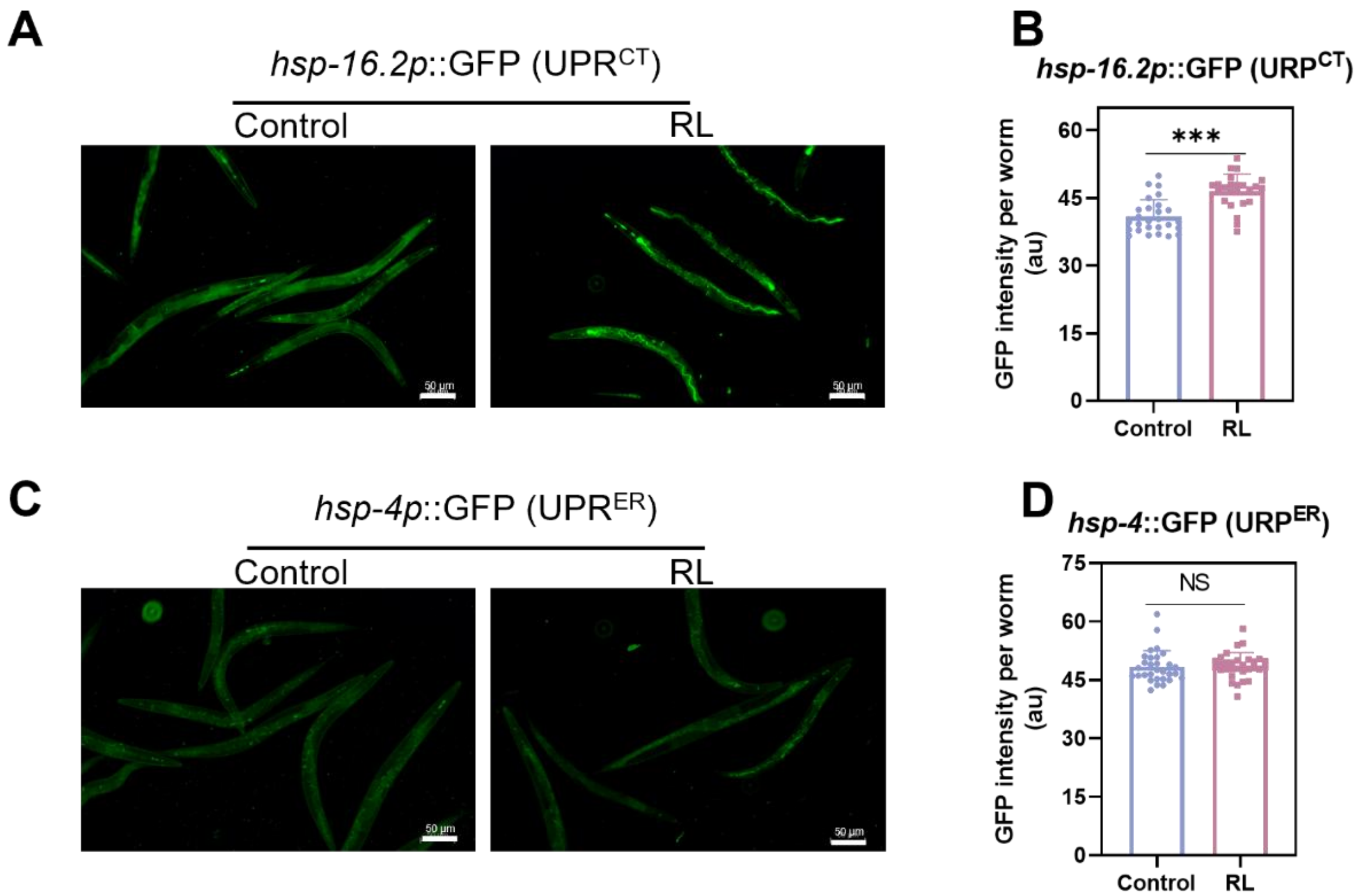

Supplementary Figure 8. Effect of red light on the response of cytoplasmic and endoplasmic reticulum unfolded proteins. (A, B), Representative fluorescence images (A) and quantification (B) of *Phsp-16.2::GFP* expression in the cytosol of transgenic worms (TJ375) after red light exposure (n = control: 27; RL: 24; Scale bar, 50 μm). (C, D), Representative fluorescence images (C) and quantification (D) of *Phsp-4::GFP* expression in the endoplasmic reticulum of transgenic worms (SJ4005) after red light exposure (n = control: 30; RL: 31; Scale bar, 50 μm). All assays were independently repeated three times. "n" indicates the number of individual worms per group (biological replicates). Data are mean ± SD. Statistical significance was determined using two-sided Student's *t*-test. NS, not significant; *** *P* < 0.001 *vs* Control.

© 2025. Zhu Y et al. Published online at http://www.aginganddisease.org/EN/10.14336/AD.2025.0791

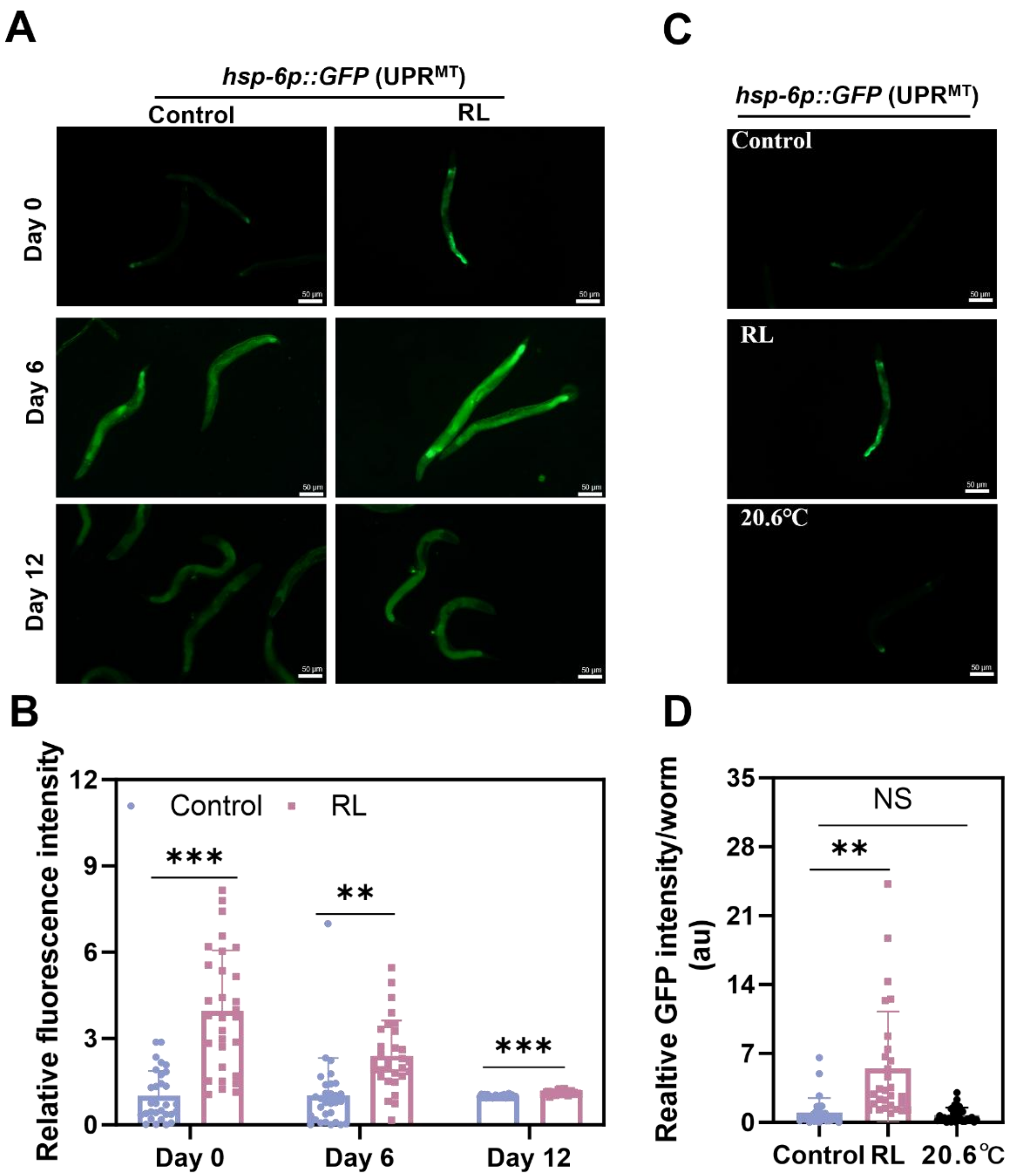

Supplementary Figure 9. Sustained activation of the mitochondrial unfolded protein response (UPR^{MT}) following early-life red light exposure, with temperature effects excluded. Representative fluorescence images (A) and quantification (B) of *Phsp-6::GFP* transgenic worms on days 0, 6, and 12 after red light exposure (A), relative fluorescence intensity compared with controls (B) ((n = control: 30 (day 0), 30 (day 6), 23 (day 12); RL:30 (day 0), 30 (day 6), 29 (day 12); Scale bar, 50 μm). (C), Representative fluorescence images and quantification of Phsp-6::GFP compared with dark control on day 0 after red light exposure or a 0.6°C temperature increase compared with dark control (n = 29 per group; Scale bar, 50 μm). All assays were independently repeated three times. "n" indicates the number of individual worms per group (biological replicates). Data are mean ± SD. Statistical significance was determined using two-sided Student's *t*-test. NS, not significant; $^{**}P < 0.01$, $^{***}P < 0.001$ *vs* Control.

© 2025. Zhu Y et al. Published online at http://www.aginganddisease.org/EN/10.14336/AD.2025.0791

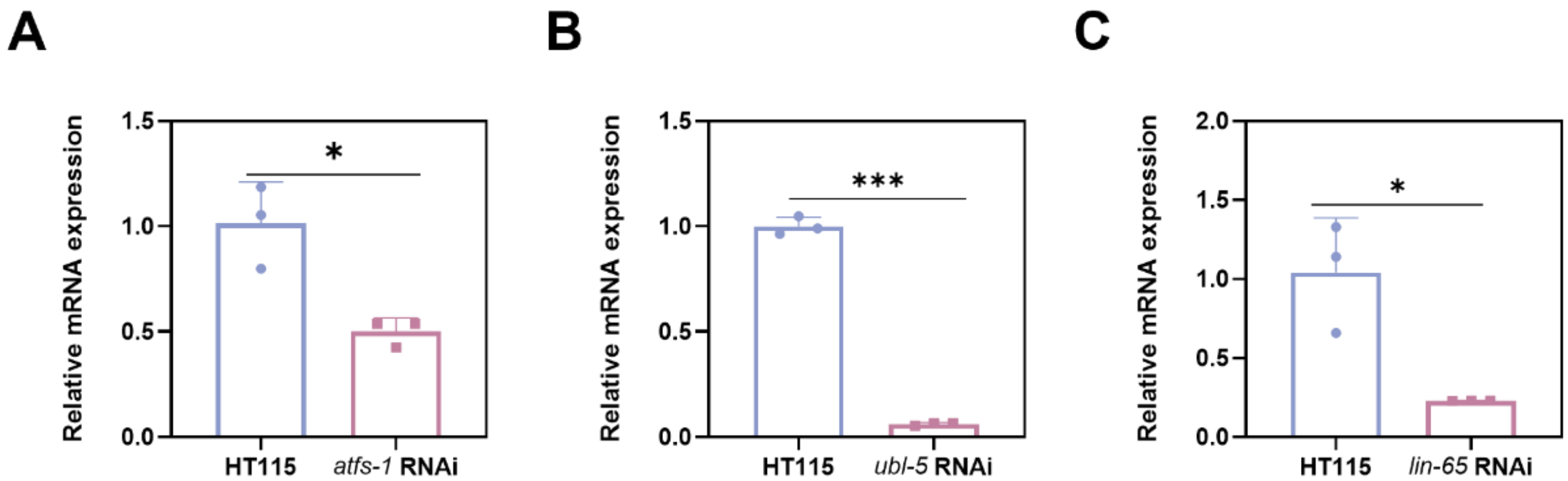

Supplementary Figure 10. Validation of RNAi knockdown efficiency. (A–C), Relative mRNA expression of *atfs-1* (A), *ubl-5* (B), and *lin-65* (C) following RNAi-mediated knockdown in WT worms relative to L4440 empty-vector controls. Worms were fed bacteria expressing dsRNA targeting the indicated genes from the L1 stage, and samples were collected at the young adult stage for RT–qPCR analysis. All experiments were conducted at 20°C on NGM plates seeded with either control bacteria (empty vector) or bacteria expressing dsRNA targeting *atfs-1, ubl-5,* or *lin-65.* All assays were independently repeated three times. Data are mean ± SEM. Statistical significance was determined using two-sided Student's *t*-test. NS, not significant; * $P < 0.05$; *** $P < 0.001$ *vs* HT115.

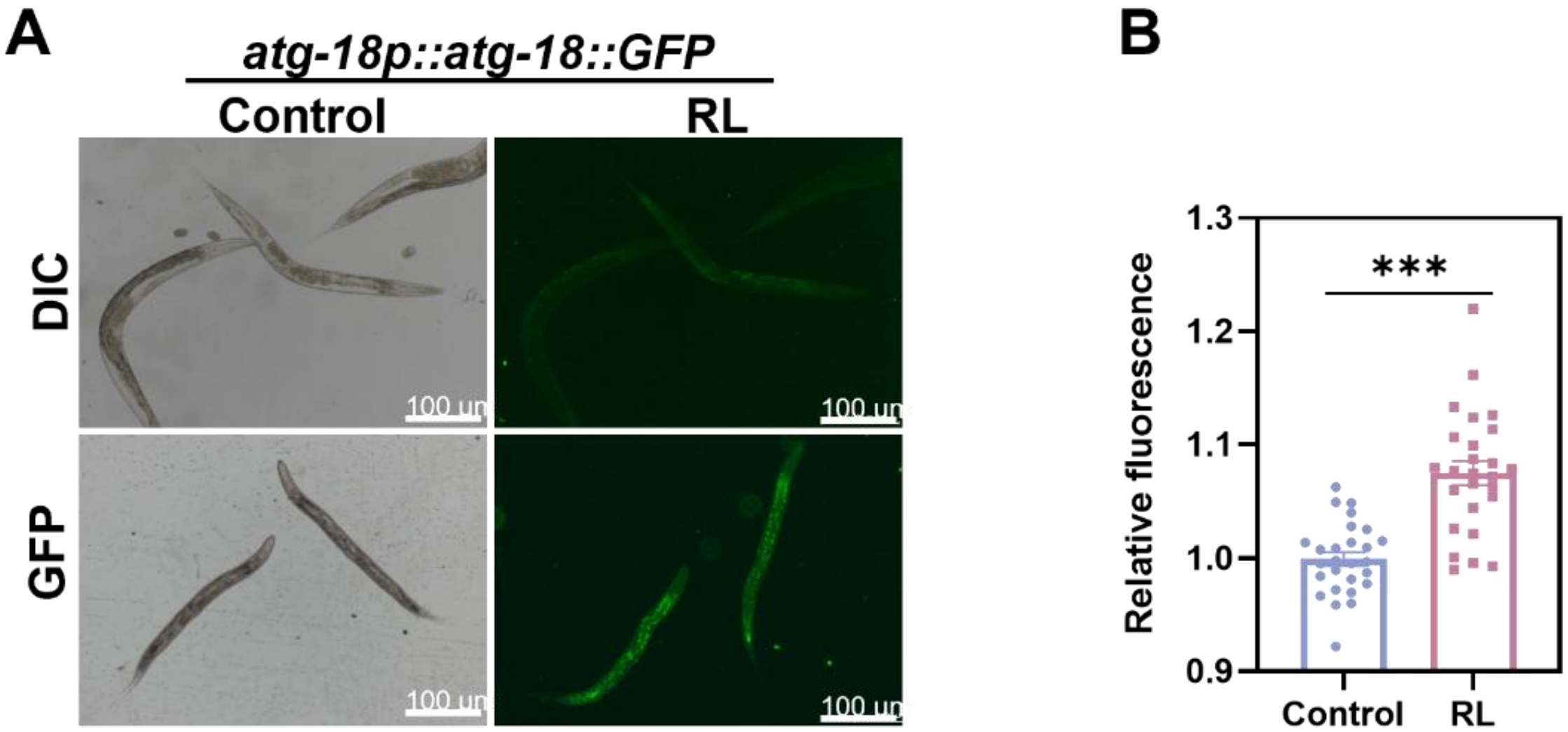

Supplementary Figure 11. ATG-18 activation in response to early-life red light exposure. (A, B), Representative fluorescence images (A) and quantification (B) of *atg-18p::GFP* intensity compared with control (n = control: 27; RL: 26; scale bar, 100 µm). Assay was independently repeated three times. Data are mean ± SD. Statistical significance was determined using two-sided Student's *t*-test. *** $P < 0.001$ *vs* Control.

© 2025. Zhu Y et al. Published online at http://www.aginganddisease.org/EN/10.14336/AD.2025.0791

SUPPLEMENTARY DATA

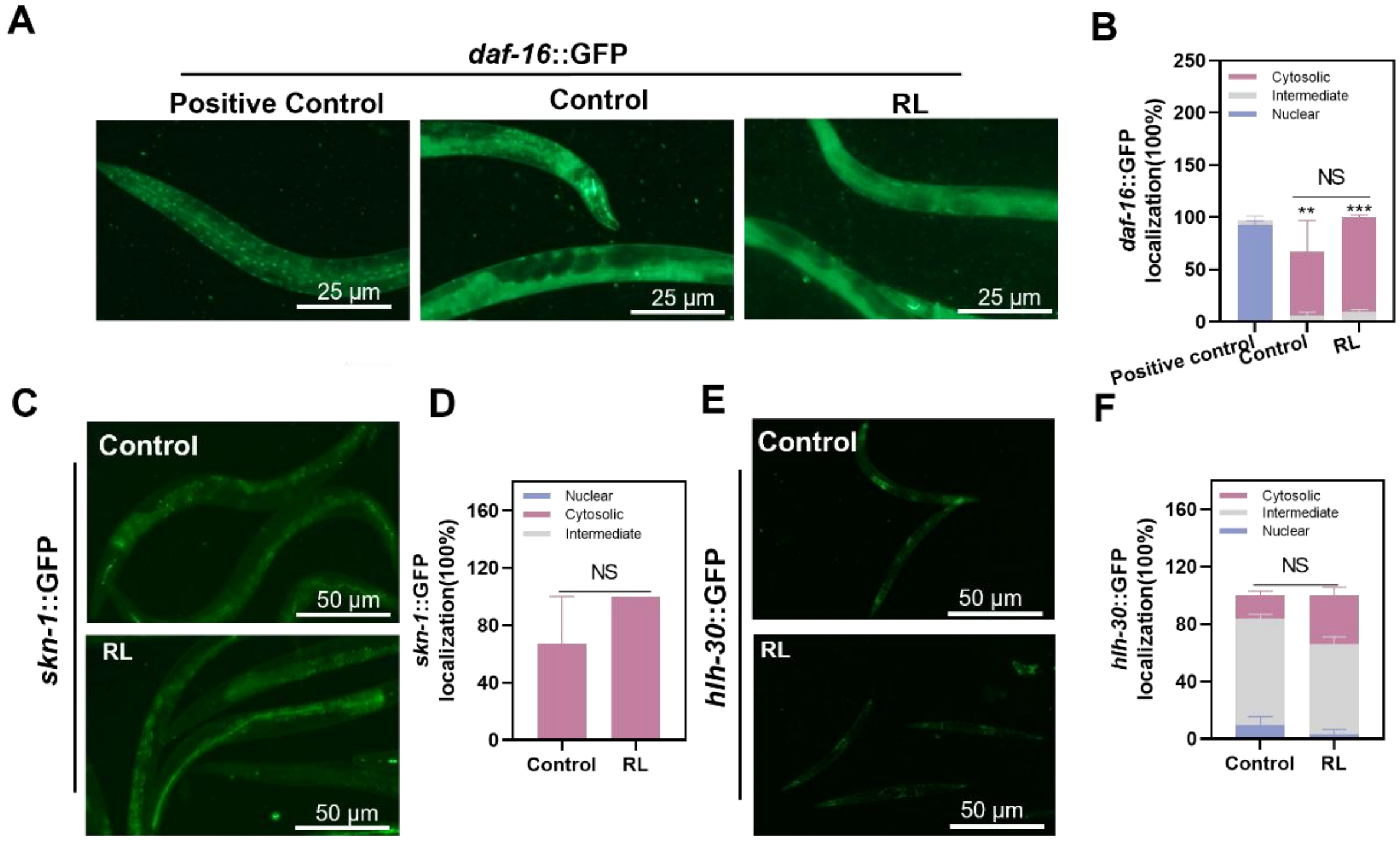

Supplementary Figure 12. The effect of red light on activating key transcription regulatory factors of mitophagy. (A, B), Representative fluorescence images (A) and quantification (B) of *daf-16p::daf-16a/b::GFP* expression in TJ356 worms after early-life red light exposure or 1-hour heat stress at 37 °C (n = 30 (positive control), 28 (control), 31 (RL); Scale bar, 50 μm). (C, D), Representative fluorescence images (C) and quantification (D) of *skn-1b/c::GFP* expression in LD1 worms after early-life red light exposure (n = control: 60; RL: 40; Scale bar, 50 μm). (E, F), Representative fluorescence images (E) and quantification (F) of *hlh-30p::hlh-30::GFP* expression in MAH235 worms after early-life red light (n = control: 43; RL: 38; Scale bar, 50 μm). All assays were independently repeated three times. "n" indicates the number of individual worms per group (biological replicates). Data are mean ± SD. Statistical significance was determined using one-way ANOVA followed by Dunnett's multiple comparison test. NS, not significant.

© 2025. Zhu Y et al. Published online at http://www.aginganddisease.org/EN/10.14336/AD.2025.0791

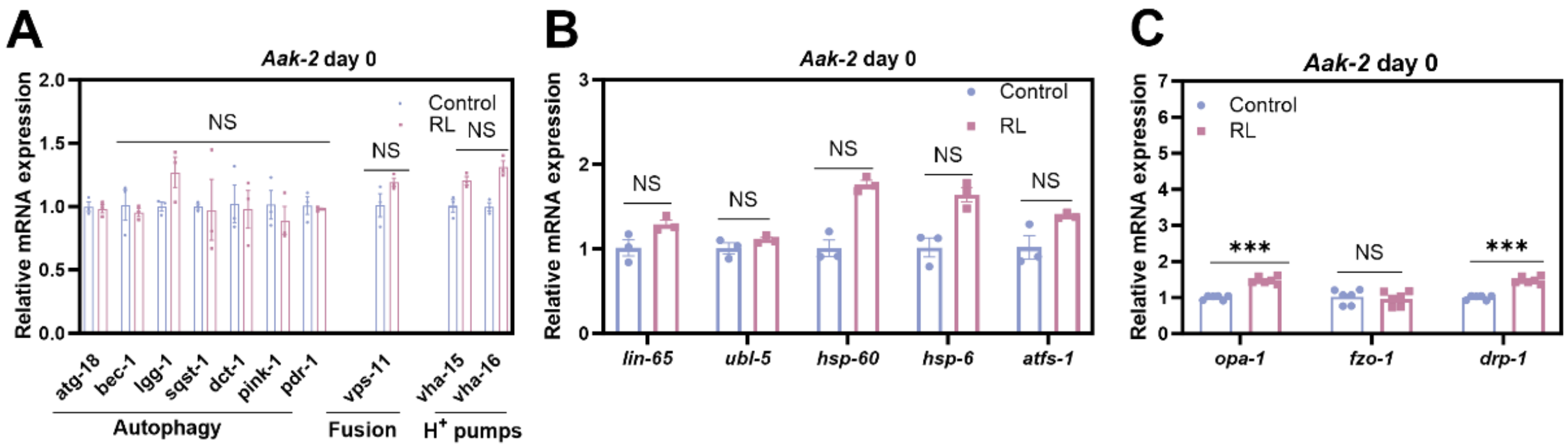

Supplementary Figure 13. Loss of AMPK abolishes red-light-induced transcriptional activation of key mitochondrial quality control genes. (A- C), Relative mRNA expression of representative genes involved in (A) mitophagy, (B) the mitochondrial unfolded protein response, and (C) mitochondrial dynamics in *aak-2(ok524)* mutants following early-life red-light exposure. Relative mRNA levels were normalized to *act-1* and expressed as fold change relative to non-treated mutant controls. All assays were independently repeated three times. Data are mean ± SEM. Statistical significance was determined by two-sided Student's *t*-test. NS, not significant, *** *P* < 0.001 *vs* Control.

© 2025. Zhu Y et al. Published online at http://www.aginganddisease.org/EN/10.14336/AD.2025.0791

SUPPLEMENTARY DATA

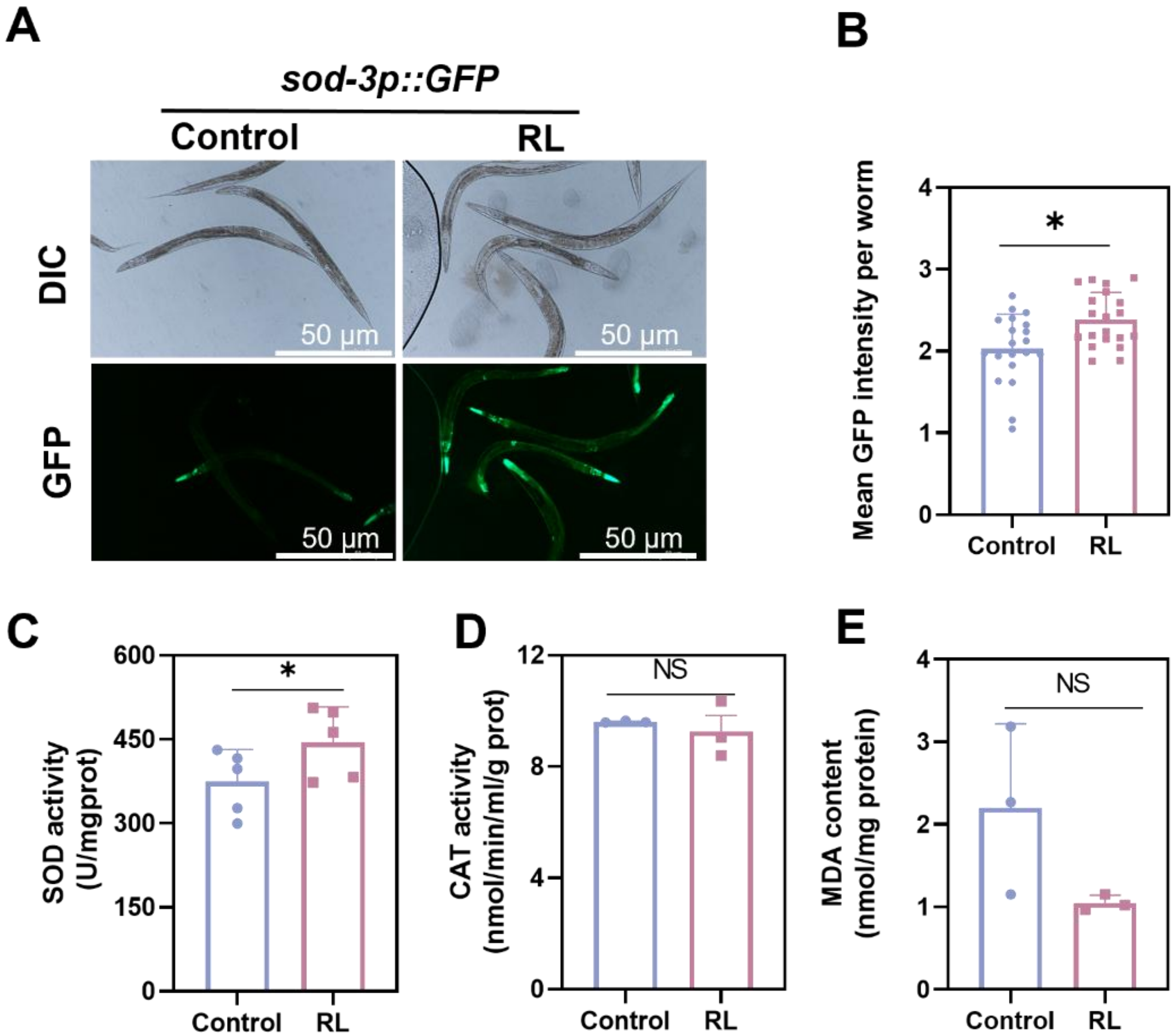

Supplementary Figure 14. Red light exposure activates antioxidant responses without causing oxidative damage in *C. elegans.* (A, B), Representative fluorescence images (A) and quantification (B) of *sod-3p::GFP* expression in CF1553 worms after early-life red light exposure (n = 20 per group; Scale bar, 50 μm). (C–E), Quantification of endogenous antioxidant enzyme activity (SOD and catalase) and lipid peroxidation marker (MDA) in WT worms after early-life red light exposure. SOD and catalase activities are expressed as U/mg protein. Assays were independently repeated three times (A, B, D, E); panel C represents five independent biological replicates. "n" indicates the number of individual worms per group (biological replicates). Data are mean ± SD (B) or mean ± SEM (C–E). Statistical significance was determined by two-sided Student's *t*-test. NS, not significant; * $P < 0.05$ *vs* Control.

© 2025. Zhu Y et al. Published online at http://www.aginganddisease.org/EN/10.14336/AD.2025.0791

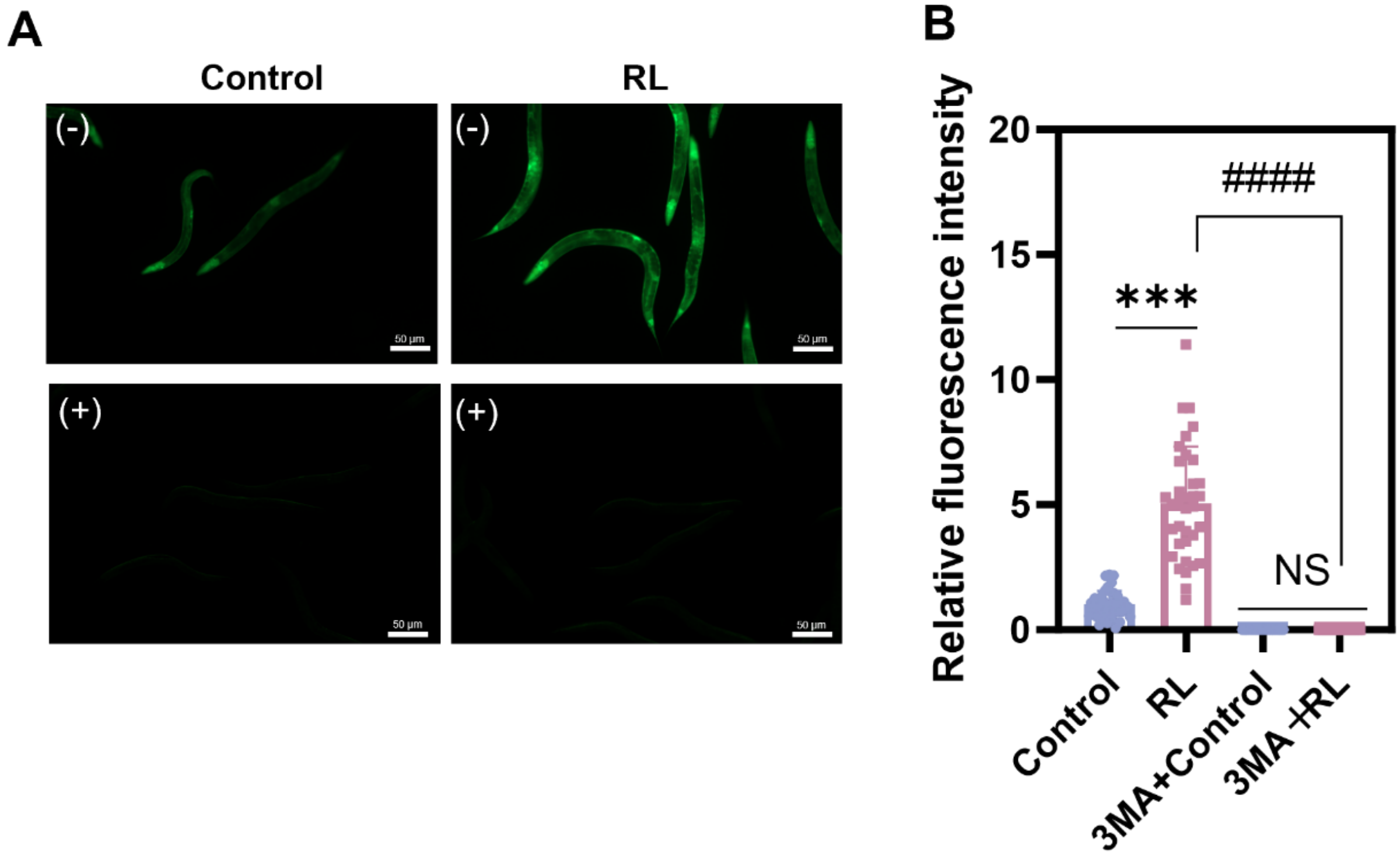

Supplementary Figure 15. Autophagy inhibition attenuates red-light-induced AMPK activation in *C. elegans.* (A, B), Representative fluorescence images (A) and quantification (B) of *aak-2p::aak-2::GFP* expression in the transgenic reporter strain WBM60 [aak-2p::aak-2(1–321)::GFP+myo-2p::tdTomato] following early-life exposure to red light in the presence or absence of the autophagy inhibitor 3-methyladenine (3-MA, 10 mM). L1-young adult stage worms were treated as indicated, and fluorescence was visualized by epifluorescence microscopy (n = control:37, RL:35, 3-MA+ control:32, 3-MA+ RL:30; Scale bar, 50 μm). "n" indicates the number of individual worms per group (biological replicates). Data are mean ± SD. Statistical significance was determined by two-sided Student's *t*-test. NS, not significant; *** $P < 0.001$ *vs* Control; $^{\#\#\#}$ $P < 0.001$ *vs* RL.

© 2025. Zhu Y et al. Published online at http://www.aginganddisease.org/EN/10.14336/AD.2025.0791

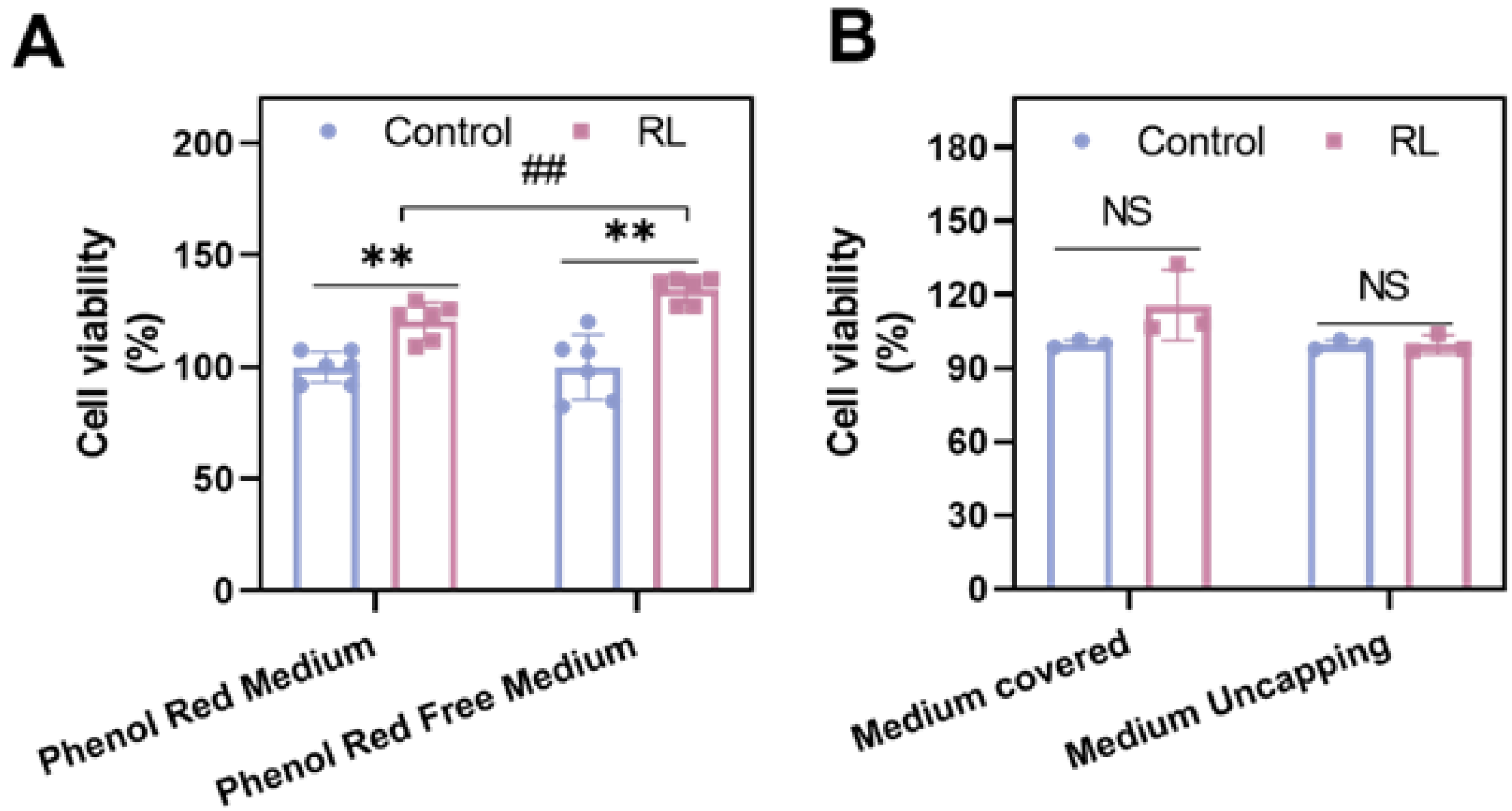

Supplementary Figure 16. Effect of DMEM medium composition and culture dish lid presence on cell viability under red light exposure. (A), Cell viability of HFF-1 cells exposed to red light for 4 h, comparing DMEM with or without phenol red, assessed using the CCK-8 assay. (B), Cell viability of HFF-1 cells cultured in phenol red-free DMEM and exposed to red light for 4 h with or without the culture dish lid, assessed using the CCK-8 assay. All assays were independently repeated three times. Data are mean ± SEM. Statistical significance was determined using two-sided Student's *t*-test. NS, not significant; ** $P < 0.01$ vs Control. $^{\#\#}$ $p < 0.01$ vs Phenol red-containing medium group.

© 2025. Zhu Y et al. Published online at http://www.aginganddisease.org/EN/10.14336/AD.2025.0791

SUPPLEMENTARY DATA

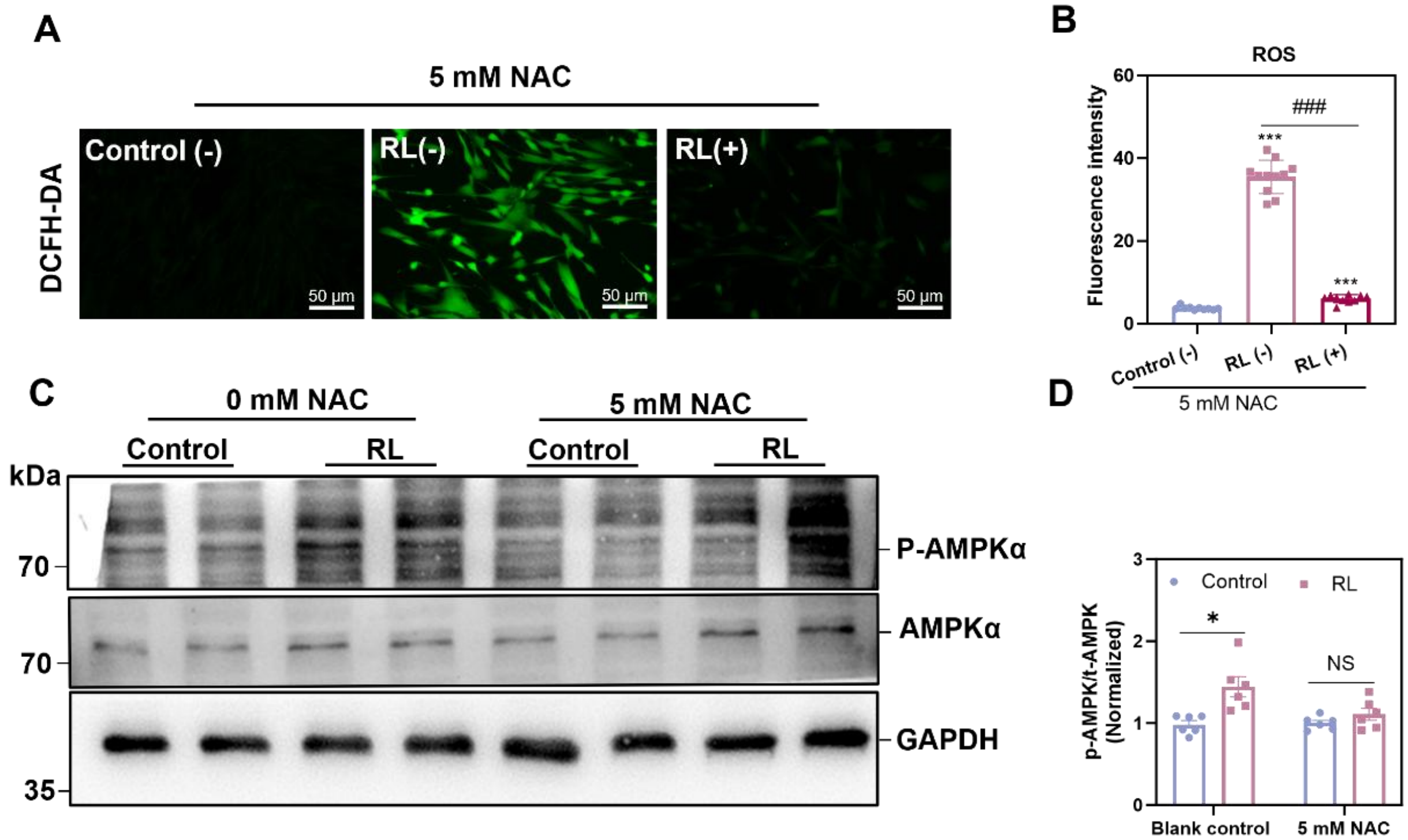

Supplementary Figure 17. ROS scavenging by NAC abolishes red-light-induced AMPK activation in human fibroblasts. (A, B) Representative fluorescence microscopy images (A) and quantification (B) of intracellular ROS levels in HFF-1 cells after 4 h of red-light (RL) exposure, with or without the ROS scavenger N-acetylcysteine (NAC, 5 mM) (n = control (-): 10, RL (-): 11, RL (+): 10; Scale bar, 50 μm). (C, D), Western blot analysis of total AMPK and phosphorylated AMPK (p-AMPK) levels in the same treatment groups, with densitometric quantification in (D). Protein expression was normalized to GAPDH and the p-AMPKα/AMPKα ratio calculated (n=6 per group). All assays were independently repeated three times. For fluorescence quantification (panel B), "n" denotes the number of independent images from distinct biological replicates, whereas for Western blot quantification (panel D), n = 6 refers to six independent biological samples. Data in (B) are shown as mean ± SD, and data in (D) are shown as mean ± SEM. Statistical significance was determined using two-sided Student's *t*-test. NS, not significant; *** $P < 0.001$ vs control; $^{###}$ $p < 0.001$ *vs* RL without NAC.

References

[1] Zhao C, Fu K, Tian J, Long T, Song J, Chen S, et al. (2025). Wearable photobiomodulation halts thyroid cancer growth by leveraging thyroid photosensitivity. Bioeng Transl Med, 10:e10734.

© 2025. Zhu Y et al. Published online at http://www.aginganddisease.org/EN/10.14336/AD.2025.0791

SUPPLEMENTARY DATA

© 2025. Zhu Y et al. Published online at http://www.aginganddisease.org/EN/10.14336/AD.2025.0791
